# Distributional Characterization of ICV-Normalized Regional Brain Volumes in Alzheimer’s Disease †

**DOI:** 10.3390/life16101741

**Published:** 2026-10-16

**Authors:** Hamsha Varsha Somangalin Ramanathan, Charles Thompson, Tarun Goswami

**Affiliations:** 1 Department of Biomedical, Industrial and Human Factors Engineering, Wright State University, 3640 Colonel Glenn Hwy, Dayton, OH 45435, USA; somangalinramanathan.2@wright.edu; 2 Boonshoft School of Medicine, Wright State University, 3640 Colonel Glenn Hwy, Dayton, OH 45435, USA; thompson.853@wright.edu

**Keywords:** Alzheimer’s disease, ADNI, MRI, distribution fitting, exponentiated Weibull distribution, maximum likelihood estimation, intracranial volume normalization, regional brain volume, model adequacy, goodness of fit, bootstrap

## Abstract

**Introduction:** Alzheimer’s disease (AD) has progressive structural brain changes that can be described using magnetic resonance imaging (MRI). Previous work has used several statistical approaches to compare the volume of brain regions such as the hippocampus, entorhinal cortex, and amygdala. However, many of these approaches assume a Normal or symmetric distribution of regional volumes, which may not fully capture the variance and regional morphological variability of observed brain volumes. This study compares different probability distributions to assess the distributional flexibility needed to characterize brain volumes normalized to intracranial volume (ICV). **Method:** Six candidate probability distributions were evaluated across 21 FreeSurfer-derived, ICV-normalized regional brain volumes classified by sex and diagnosis group (Female-CN, Male-CN, Female-AD, and Male-AD). After ICV quality control, data were drawn from 3409 T1-weighted 3T MRI scans of 1343 participants in the Alzheimer’s Disease Neuroimaging Initiative (ADNI); the primary analysis used one baseline scan per participant. The resulting 84 region-by-group combinations (21 regions × 4 groups) were fitted using maximum likelihood, with all four parameters of the four-parameter Exponentiated Weibull (Weibull4P) estimated by multistart, ridge-stabilized maximum likelihood with identifiability safeguards. Distributions were compared with the Akaike information criterion (AIC), its small-sample correction (AICc) and the Bayesian information criterion (BIC). A distribution was regarded as adequate relative to other candidates when the AIC was within 10 units of the lowest AIC. Absolute fit was assessed with parametric bootstrap goodness-of-fit tests and robustness was assessed with a participant-level bootstrap, five-fold cross-validation of all six distributions and age-stratified refits. **Results:** The Normal distribution gave the lowest AIC in 40 of the 84 combinations, Gamma in 21, Lognormal in 12, Weibull4P in 10 and the two-parameter Weibull in one. Two or more distributions were within 2 AIC units of the lowest AIC in 51 combinations. Weibull4P was adequate by the relative AIC (ΔAIC < 10) in all 84 combinations, compared with 74 for Gamma, 68 for Normal and 66 for Lognormal; its largest difference from the lowest AIC was 4.9. In the 12 ventricular combinations, Normal was adequate in only one, compared with all 12 for Weibull4P. Weibull4P retained relative AIC support in at least 95% of the 500 participant-bootstrap replicates in 83 combinations and in all three age bands in every combination, including refits that did not meet StablePass. It met the descriptive BIC threshold in 53 combinations and the cross-validation threshold in 55. In the parametric-bootstrap goodness-of-fit tests, Weibull4P was not rejected by the uncorrected Anderson–Darling test in 71 of the 84 combinations, more than any other candidate (Gamma: 60; Normal: 57). Normality was rejected after FDR correction in 21 combinations, including 10 ventricular combinations. Of the 42 sex-specific AD–CN comparisons, 41 were significant after age adjustment and FDR correction. **Conclusions**: The Normal distribution most often had the lowest AIC and had relative support in most tissue combinations in this ADNI dataset, but rarely in ventricular combinations. The four-parameter Exponentiated Weibull had the lowest AIC in fewer combinations but was the only candidate with relative AIC support across all regions and groups, including the right-skewed ventricular volumes. It was also rejected less often by the Anderson–Darling goodness-of-fit test than any other candidate. However, its additional parameters were less supported by BIC, and its estimated lower bound restricted its out-of-sample performance. These findings support an empirical approach to evaluating distributional form rather than assuming normality, with Exponentiated Weibull as an alternative to consider alongside simpler distributions.

## 1. Introduction

Alzheimer’s disease (AD) is the most prevalent type of dementia and is an important public health issue. An estimated 7.4 million people aged 65 and older are living with clinical AD in the United States in 2026, and this figure is expected to rise to 13.8 million by 2060 in the absence of medical breakthroughs [1]. Worldwide, it is estimated that over 57 million people have dementia [2]. Thus, there is a need for reliable and well-characterized quantitative biomarkers that can facilitate the early detection of AD, assessment of disease severity, and monitoring of disease progression and treatment response. Magnetic resonance imaging (MRI) is a commonly used, non-invasive imaging method that can provide quantitative information about brain structure in vivo. Brain volume, particularly in regions associated with AD-related neurodegeneration such as the hippocampus, entorhinal cortex, and amygdala, has been examined as a potential indicator of AD. Brain volumes are commonly normalized to intracranial volume (ICV) to account for differences in overall head and brain size between individuals [3,4]. Using FreeSurfer-derived ADNI volumes, previous work from our group reported that ICV-normalized medial temporal volumes differed more between AD and CN adults than global gray and white matter volumes when analyzed with linear mixed-effects models [5] and that MRI-derived structural estimates can depend on voxel geometry and the segmentation method used [6]. Researchers then often compare these normalized measurements across diagnostic groups using statistical methods such as *t*-tests, analysis of covariance (ANCOVA), mixed-effects models, and normative percentile methods. Many of these methods rely on normal-theory statistics and/or the assumption that morphometric measurements in the underlying population follow a Normal distribution. Neuroimaging measures may depart from Normal distributions, and flexible non-Gaussian models have therefore been proposed for normative modeling [7]. Atrophy may occur differently across different phases of the disease, different pathologies, and different brain regions that are more susceptible to neurodegeneration [8]. Brain-volume measurements are also naturally limited, as a region can have a volume of zero or greater, and there is an upper limit on a region’s volume based on the number of tissues available. These properties, as well as inter-group and inter-regional variation, can lead to non-normal distributions. Because of this, examining the underlying distribution of regional brain volumes rather than assuming a Normal distribution may provide a more appropriate characterization of these measurements. Several probability distributions can be used when data are not normally distributed. One example is the Exponentiated Weibull distribution proposed by Mudholkar and Srivastava [9]. The Exponentiated Weibull distribution is an extension of the standard Weibull distribution that includes an additional shape parameter, enabling it to describe densities that are monotonically decreasing or unimodal, together with hazard rates that are monotone, unimodal, or bathtub-shaped [9,10]. This can be useful when biological measurements have different distributional shapes across populations or anatomical regions. Although Exponentiated Weibull distributions have been used in other applications, to our knowledge their use for describing neuroimaging morphometric measurements has not been extensively investigated. In contrast, conventional distributions such as the Normal and Lognormal distributions have been more frequently used to describe regional brain volumes. More flexible probability distributions may provide additional insight into the distributional characteristics of ICV-normalized brain volumes. In the current study, six potential distributions were tested: Normal, Lognormal, Gamma, Exponential, two-parameter Weibull, and four-parameter Exponentiated Weibull distributions. These distributions were analyzed across 21 ICV-normalized brain regions and four sex-by-diagnosis groups. The two-parameter distributions provide relatively simple models for the observed measurements, while the four-parameter Exponentiated Weibull distribution offers greater flexibility in describing the shape and location of the distribution. The comparison was therefore used to assess whether the Exponentiated Weibull, with its greater flexibility, could provide an adequate description of regional brain-volume measurements across regions and groups in which the simpler distributions may differ in their performance. The present study had three main objectives. First, we aimed to explore the distribution of ICV-normalized brain volumes in each brain region and sex-by-diagnosis group without assuming normality beforehand. Second, we examined whether the four-parameter Exponentiated Weibull distribution provided an adequate description of every region-by-group combination, for both tissue and ventricular regions, alongside the commonly assumed Normal distribution and whether its two additional parameters were supported by the data. Third, we described whether the distributional form differed between cognitively normal (CN) and AD groups and quantified AD-CN differences in age-adjusted regional volumes within each sex. We hypothesized that, in addition to the Normal distribution, the four-parameter Exponentiated Weibull would provide an adequate description of ICV-normalized volumes in both tissue and ventricular regions. In this study, the lowest-AIC distribution in a combination is the one with the smallest AIC and a distribution is regarded as adequate when its AIC is within 10 units of the lowest AIC; a distribution can therefore be adequate without having the lowest AIC. Because several candidate distributions can fit a given sample almost equally well, the study focused on whether each distribution was adequate in each combination rather than only on which distribution had the lowest AIC.

After the first stage of the analysis, which compared the six distributions, subsequent analyses examined how much confidence could be placed in these comparisons. Because many ADNI participants contributed more than one scan, the analysis was performed at the participant level, using one baseline scan per participant. The absolute fit of each of the six distributions was examined with parametric bootstrap goodness-of-fit tests; the stability of the result was examined by resampling participants; the out-of-sample performance of all six distributions was examined by five-fold cross-validation and the dependence of fits on age was examined by refitting the distributions within age bands. This multistage evaluation enabled us to examine whether greater distributional flexibility provided an adequate description of regional MRI morphometric measurements in AD, while also quantifying the uncertainty associated with the comparison of candidate distributions.

## 2. Materials and Methods

### 2.1. Data Source and Participants

The ADNI database was used for data acquisition in this study (adni.loni.usc.edu, accessed on 1 July 2026). ADNI began in 2003 as a public–private partnership led by Dr. Michael W. Weiner to determine whether serial MRI, PET, other biological measures, and clinical and neuropsychological evaluations could be used together to monitor the course of mild cognitive impairment (MCI) and early Alzheimer’s disease. Current goals involve trying to improve the generalizability of ADNI-collected data by increasing diversity in the cohort, providing data concerning AD-related pathology, and improving clinical biomarkers [11]. The large sample size and diagnoses in ADNI allowed us to estimate the distributional characteristics of different brain regions across a broad population, rather than relying on the smaller sample size of a single cohort. The source data included brain-volume measurements for individual regions derived from MRI scans, along with age, sex, and AD diagnosis for each participant.

This study divided participants into two diagnostic groups: cognitively normal (CN) and Alzheimer’s disease (AD). After the ICV quality control described in Section 2.2, a total of 3409 MRI scans remained, including 2736 scans from CN individuals and 673 scans from individuals with AD. The dataset included 1343 unique participants (1055 CN and 288 AD), of whom 721 contributed more than one scan. All 3409 scans were acquired at 3T. The mean age at the baseline scan was 71.1 ± 7.8 years overall, with a mean age of 70.3 ± 7.5 years for the CN group and 74.0 ± 8.1 years for the AD group.

Many participants contributed more than one MRI scan over time, while others contributed only a single scan. Because repeated scans from the same participant are not statistically independent [12], the primary distribution fitting analysis used one scan per participant, namely the earliest available scan, referred to here as the baseline scan (defined as the ADNI screening or baseline visit in 1255 of the 1343 participants; for the three participants with two scans on the same date, the first listed record was used) so that each participant contributed at most one observation to each region-by-group fit. The longitudinal data for each participant were not averaged to a single value, and the repeated scans were not used in the distribution fitting, because scans from the same participant remain correlated.

Age was determined as (scan date – birth date)/365.25. Because the ADNI data used for this analysis reported only the birth month and year, we assigned the day of birth as the first day of the reported birth month. This introduces a small rounding error of less than one month, which is small relative to the sample’s approximate 8-year standard deviation of age. Very young and very elderly participants were underrepresented, with most participants in this study being between 60 and 80 years of age. Therefore, the results should be interpreted within the context of this age range.

### 2.2. Data Quality and Regional Volume Normalization

Values that were not successfully segmented for a given region and scan were treated as missing data rather than being coded as zero volume. These observations were excluded at the region/scan level. Treating unsuccessful segmentations as zero volumes would artificially shift the fitted distributions toward lower values and could make brain atrophy appear greater than it was. Excluding these observations resulted in slightly different sample sizes across individual regions. In the baseline (one scan per participant) data, the number of participants per region-by-group combination ranged from 621 to 666 for Female-CN, 366 to 388 for Male-CN, 127 to 133 for Female-AD and 144 to 154 for Male-AD; region-specific sample sizes are reported in Table A1. The potential influence of this region-specific missingness on the fitted distributions was not formally analyzed.

All regional brain volumes were extracted using FreeSurfer (Laboratory for Computational Neuroimaging, Athinoula A.Martinos Center for Biomedical Imaging, Charlestown, MA, USA) [13]. Two measures were obtained from the MRI scans: regional volume, measured in mm^3^, and total intracranial volume (ICV), which provides a measure of the overall size of the intracranial space. All regional brain volumes were normalized by ICV for analysis. This normalization accounts for differences in head and brain size between individuals. Without normalization, an individual with a naturally larger head may have a larger hippocampus than an individual with a smaller head despite having the same disease-related changes. Comparing raw regional volumes would therefore combine variation associated with disease-related atrophy with normal variation in head size. Dividing regional volume by ICV helps account for this source of variation. However, regional volumes do not scale exactly in proportion to head size, so proportional normalization does not remove all size-related variation; the ICV-normalized volumes analyzed here should therefore be interpreted as volume fractions rather than as fully size-independent measures. Before normalization, each scan’s FreeSurfer ICV was checked for plausibility: ICV of 1.0–2.1 × 10^6^ mm^3^ and brain-tissue fraction [(total gray matter + cortical white matter)/ICV] of 0.506–0.892. Scans were flagged for missing or implausible ICV or a >10% deviation from the participant’s median plausible ICV. Participants were flagged entirely if they had no plausible scan or fewer than three plausible scans with >15% variation. Flagged baseline participants (*n* = 50; 17 CN, 33 AD) were excluded, and flagged follow-up scans (*n* = 52) were removed without imputation, leaving 3409 of 3607 scans and 1343 of 1393 participants. The regions analyzed included global measures (cortical gray matter, total gray matter and cortical white matter), medial temporal lobe structures (hippocampus, entorhinal cortex, amygdala, and parahippocampal gyrus), ventricular structures (inferior lateral ventricle, lateral ventricle, and third ventricle), and neocortical regions (middle temporal gyrus, inferior temporal gyrus, superior temporal gyrus, precuneus, posterior cingulate, fusiform gyrus, inferior parietal lobule, superior parietal lobule, rostral middle frontal gyrus, anterior cingulate, and superior frontal gyrus). The present study did not treat the 21 regional volumes as validated diagnostic biomarkers; rather, it characterized their ICV-normalized distributions as MRI-derived anatomical measures.

### 2.3. Stratification

The observations were divided into four groups: Female-CN, Male-CN, Female-AD, and Male-AD. Sex was coded as 1 = male and 2 = female in the source data. The data were stratified by both diagnosis and sex rather than by diagnosis alone because differences in brain volume and patterns of atrophy have been reported between males and females, as well as differences in the prevalence of Alzheimer’s disease [14,15]. Therefore, sex was considered an important characteristic of the populations being evaluated rather than treated only as a nuisance variable.

Combining males and females within the same diagnostic group could mask differences in the distribution of regional brain volumes between the sexes. Stratification by sex and diagnosis therefore allowed the distribution of each brain region to be examined separately for CN and AD participants within each sex. This approach also allowed us to determine whether the distribution selected for a specific brain region was consistent between males and females and between diagnostic groups. This was directly related to the third objective of the study, which described whether the selected distributional form differed between diagnostic groups and sexes. Because the four groups were fitted separately, such comparisons are descriptive; no formal sex × diagnosis interaction test of distributional shape was performed.

### 2.4. Candidate Distributions

The study fitted six candidate probability distributions to the ICV-normalized volume data separately for each of 21 brain regions and each of the 4 demographic groups (Female-CN, Male-CN, Female-AD and Male-AD), resulting in 21 × 4 = 84 region-by-group distributional analyses using the baseline scan for each participant. Before fitting, we removed any invalid values (missing, infinite, or zero or negative volumes) and excluded any groups with fewer than 10 valid data points, though every region × group combination in our data had enough observations to include. The six candidate distributions, together with their parameters and estimation method, are summarized in Table 1.

The first five distributions were fitted using the built-in fitdist function of the MATLAB R2026a (The MathWorks, Inc., Natick, MA, USA) Statistics and Machine Learning Toolbox, which gives maximum likelihood estimates, except that the standard deviation parameters of the Normal and Lognormal distributions are computed with the n − 1 divisor. The four-parameter Exponentiated Weibull distribution, also referred to as Weibull4P, was not available in the standard toolbox and was therefore coded manually based on the family of distributions originally proposed by Mudholkar and Srivastava [9]. The two-parameter Weibull distribution has a shape parameter (B) that modifies the shape and skewness of the distribution and a scale parameter (A) that stretches or compresses the distribution curve. The Exponentiated Weibull distribution has two additional parameters: a location threshold parameter (loc), which shifts the lowest bound of the support of the distribution, and a second shape parameter (a), which is not present in the original two-parameter Weibull distribution. This additional shape parameter provides greater flexibility for describing more complex and less symmetric distributional patterns than can be described using the two-parameter Weibull. In the final implementation, all four parameters (a, b, c, and loc) were estimated by maximum likelihood and k = 4 was therefore used for Weibull4P in the AIC and BIC calculations. Because loc was estimated rather than fixed, it should be interpreted as a fitted lower support parameter of the statistical model rather than as a physical minimum brain volume; in 44 of the 84 fits, the estimated loc was below zero, which is a property of the fitted model and does not imply that negative volumes are plausible. Table 2 gives the probability density function and cumulative distribution function for all six distributions. For the Exponentiated Weibull distribution, z = (x − loc)/b is used as shorthand notation.

The Exponentiated Weibull distribution is not a built-in distribution in MATLAB, so a maximum likelihood fitting procedure was developed for the four parameters, using numerical optimization (fminunc). The optimization was not performed from a single initial value. Instead, a starting point based on the data was used to obtain an initial fit, followed by further optimization from 40 starting points to check that the same fit was obtained. This was important because a flexible, multi-parameter distribution can have local optima in its likelihood surface that provide a numerically good fit but do not represent the global optimum, and because maximum likelihood estimation of a location (threshold) parameter is a nonregular problem in which the usual asymptotic properties of the estimators may not hold [16]. This possibility was checked by repeating the optimization from different starting points: the multistart solutions were clustered and the proportion of starts converging to the highest likelihood solution, whether the Hessian at the solution was positive definite and whether any parameter lay on the boundary of its search range, were recorded for every fit, together with 95% profile likelihood confidence intervals for the two shape parameters (a and c). A boundary solution was not automatically accepted, because it could indicate that the optimization had not fully converged and that the resulting solution may therefore have limited value when compared with the other distributions in the subsequent analysis.

The Weibull4P likelihood can have a flat ridge along which the Weibull scale and shape parameters change together with little change in likelihood, so an L2 penalty was applied to the negative log-likelihood on the logarithms of the two shape parameters (log a and log c) with the data standardized before fitting, to obtain an estimate for each fit. The penalty weight (λ) was chosen as the smallest value in the grid {0, 0.01, 0.03, 0.1, 0.25, 0.5, 1, 2, 4, 8} that gave an identifiable solution (at least 80% of the starting points converging to the same solution, a positive definite Hessian and no boundary solution) whose unpenalized log-likelihood was within 1.92 units (95% likelihood region) of the unpenalized optimum. In 69 of the 84 combinations, λ = 0 (unpenalized maximum likelihood) was selected, and no selected value was larger than 0.03; the largest resulting increase in AIC relative to the unpenalized optimum was 0.31 units. AIC and BIC for Weibull4P were computed from the unpenalized log-likelihood evaluated at the stabilized estimate (k = 4), and the AIC of the fully unpenalized (λ = 0) multi-start fit was retained for comparison (Supplementary Table S1). The multiple-start solutions were stability-qualified (StablePass) only if the optimizer converged, at least 80% of the starting points reached a common optimum, the Hessian at the optimum was positive definite, no parameter lay on the boundary of its search range and the likelihood criterion above was met. Weibull4P was eligible to be the lowest-AIC distribution only if it passed the StablePass criterion; if an unstable Weibull4P fit had the lowest AIC or BIC, the next eligible distribution (the one with the next lowest value of the criterion) was used as the reference for that combination.

### 2.5. Model Comparison

Several criteria were used to compare the candidate distributions rather than relying on a single criterion. The main criterion used was the Akaike Information Criterion (AIC) [17], which favors a model that fits the data well while also accounting for the number of parameters. This penalty was particularly important in the present study because the Exponentiated Weibull distribution has two additional parameters to estimate compared with most other distributions considered. A less restrictive model may better fit variation or noise in the data without necessarily better representing the underlying population structure. AIC therefore allowed both model fit and model complexity to be considered. The effect of the model complexity penalty was also investigated by calculating BIC [18] and the small-sample corrected AIC (AICc) [19]. As only one baseline scan per person was used, n represents the number of persons in each region-by-group combination. ΔAIC was computed for every combination relative to the smallest AIC among the stability-qualified candidates (Section 2.4). Following Burnham and Anderson [20], distributions were deemed to have substantial support when ΔAIC < 2, while ΔAIC ≥ 10 indicated essentially no support. In this study, distributions with ΔAIC < 4 were regarded as close and those with ΔAIC < 10 as adequate relative to the other candidates. These categories refer to relative support, and absolute goodness of fit was assessed separately (Section 2.6). The same thresholds were used descriptively for the BIC differences, and agreement between distributions selected by AIC and BIC was summarized across the 84 combinations. KS and Cramér–von Mises distances between the fitted and empirical cumulative distribution functions [21] were used to rank the six distributions. Weibull4P was compared with Normal, Lognormal and Gamma using both standard and BIC-corrected Vuong tests [22], with Benjamini–Hochberg FDR correction across the 84 combinations [23]. Normality was evaluated separately for each of the 84 combinations using the Anderson–Darling test with the mean and variance estimated from the data [21,24], with FDR correction across the 84 tests, since AIC comparison is not a test of normality.

### 2.6. Goodness of Fit, Participant-Level Resampling, Cross-Validation and Age-Stratified Refitting

Absolute goodness of fit for the six distributions in all 84 region-by-group combinations was evaluated using parametric bootstrap tests [25,26], taking into account parameters estimated from the same data [27]. For each fitted distribution, 500 samples of the same size as the observed sample were simulated and refitted, and the Anderson–Darling, KS and Cramér–von Mises statistics were recomputed. Bootstrap *p*-values were calculated as (r + 1)/(B + 1), with B = 500 and r representing the number of simulated statistics at least as large as the observed statistic. For Weibull4P, the penalty weight was fixed at its observed value, and 12 starting points were used, including the observed parameter estimate. The observed statistics were obtained using the same fitting procedure. This calibration was therefore conditional on the selected penalty weight. A distribution was not rejected when *p* ≥ 0.05. No correction for multiple testing was applied to these *p*-values, because a correction would make rejection less likely and a claim of adequacy easier to meet; the uncorrected *p*-values are therefore the stricter criterion. All six distributions were assessed independently of AIC selection. Model-selection stability was assessed using 500 baseline participant-bootstrap replicates per combination, giving 42,000 replicates in total [28]. Participants, each contributing one scan, were sampled with replacement, and all six distributions were refitted, with the Weibull4P penalty weight fixed at its observed value. The proportions of replicates in which each distribution had the lowest AIC, ΔAIC < 4 and ΔAIC < 10 were recorded, together with the proportion of Weibull4P refits that passed StablePass. Unstable Weibull4P fits were excluded from selection as the lowest-AIC distribution, but their ΔAIC values were retained. Out-of-sample performance of all six distributions was assessed by participant-level five-fold cross-validation repeated twice [29], with the cross-validated deviance compared using the same thresholds as for AIC; the procedure and results are given in Supplementary Table S1. Age dependence was examined by dividing each combination into three equal-sized age bands and refitting all six distributions (252 age-band fits). Differences in volume between age bands were assessed using Kruskal–Wallis tests with FDR correction across the 84 combinations, and StablePass was recorded for each Weibull4P fit. Baseline sample sizes, likelihoods, information criteria, KS statistics, model selections, adequacy counts, normality statistics and Weibull4P–Normal Vuong statistics were independently recalculated from the raw data. Separate optimization checks assessed likelihood agreement for the remaining five distributions and whether Weibull4P estimates met the nominal 1.92-unit unpenalized log-likelihood tolerance (Supplementary Table S1). A detailed residual analysis was also performed and will be reported in a follow-up paper currently in preparation.

### 2.7. Comparison of AD and CN Groups

To place the distributional findings in a clinical context, AD–CN differences in ICV-normalized volume were estimated separately for females and males in each of the 21 regions (21 regions × 2 sexes = 42 sex-stratified comparisons), using the baseline scan of each participant. For each comparison, the unadjusted percentage difference in mean volume was calculated as [(AD mean − CN mean)/CN mean] × 100 as a descriptive measure. Because age differed between groups and is strongly associated with regional brain volume [30], the AD–CN difference was also estimated using a linear regression of ICV-normalized volume on diagnosis and age with heteroscedasticity-robust (HC3) standard errors [31], because the variance of volume differed between the diagnostic groups. The age-adjusted diagnosis coefficient was reported with its 95% confidence interval (t-distribution with n − 3 degrees of freedom), as a percentage of the CN mean and as a standardized difference (the coefficient divided by the residual standard deviation). As sensitivity analyses, the regression was repeated with log-transformed volume and an AD × age interaction term was added to examine whether the AD-CN difference varied with age. Beyond the mean, the CN and AD distributions were compared within each sex with the two-sample KS test and differences in standard deviation and skewness between the groups were estimated with bootstrap 95% confidence intervals (2000 resamples). Benjamini–Hochberg FDR correction [23] was applied across the 42 comparisons. No sex × diagnosis interaction term was fitted; comparisons of the AD–CN differences between females and males are therefore descriptive.

### 2.8. Software

MATLAB (Statistics and Machine Learning Toolbox and Optimization Toolbox) was used for model fitting, statistical comparisons, and generation of figures. Each region-by-group combination was analyzed separately for each candidate distribution.

## 3. Results

### 3.1. Sample Characteristics

The four demographic groups are summarized in Table 3. The CN group is substantially larger than the AD group in both the number of scans (2736 vs. 673) and the number of participants (1055 vs. 288). These differences reflect the composition of the analyzed ADNI dataset; AD participants also contributed slightly fewer scans on average than CN participants (2.3 vs. 2.6 scans per participant). Female-CN was the largest group (666 participants) and Female-AD the smallest (134 participants). Because each participant contributed one baseline scan, the sample size per region-by-group combination ranged from 127 to 666 participants.

### 3.2. Distribution Characteristics: The Six Model Comparison

**Figure 1:**
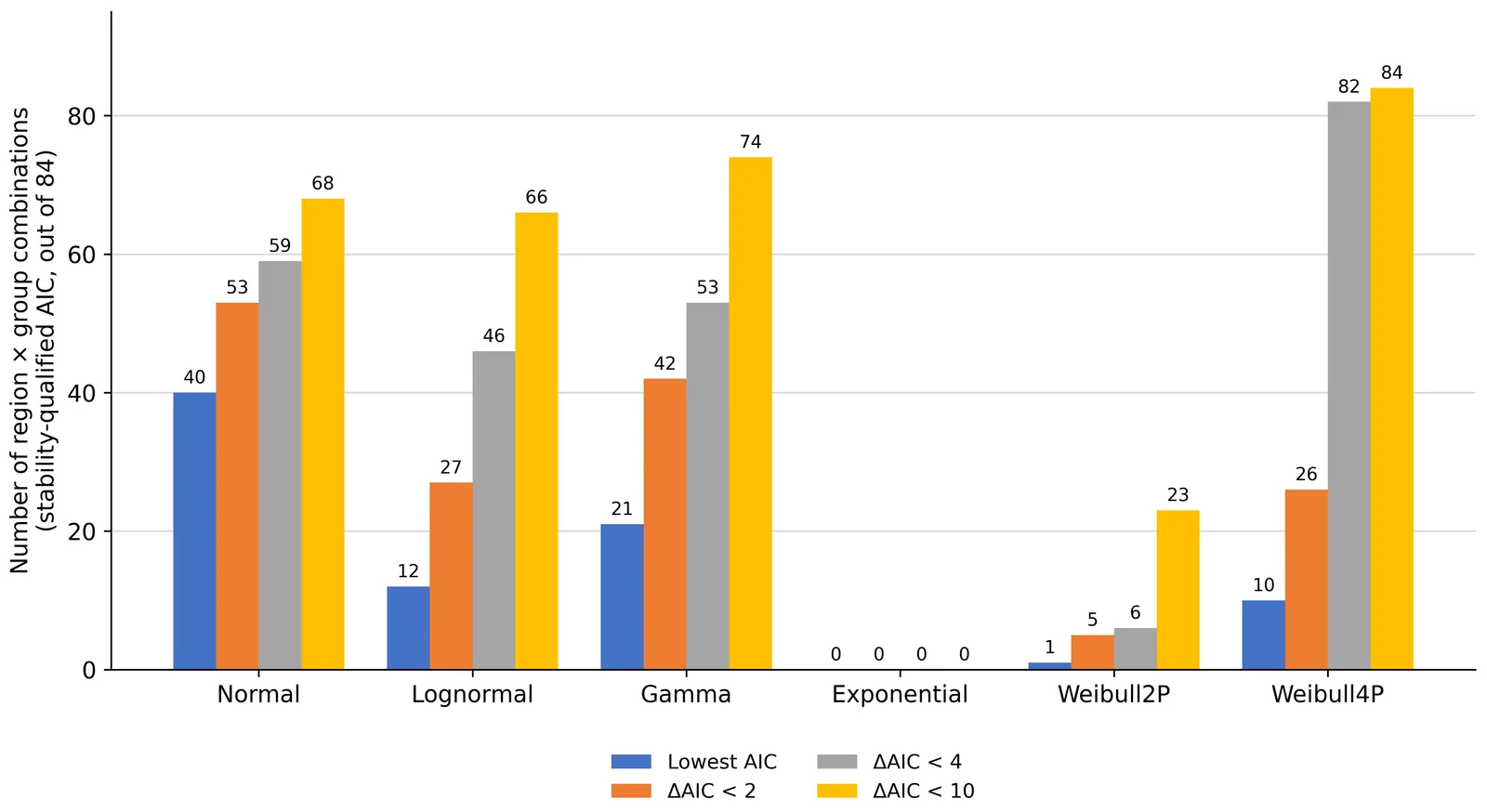
Number of the 84 region-by-group combinations (21 regions × 4 demographic groups) in which each of the six candidate distributions had the lowest AIC (ΔAIC = 0), had substantial support (ΔAIC < 2), was close (ΔAIC < 4) or was adequate (ΔAIC < 10), with ΔAIC calculated relative to the lowest AIC among the stability-qualified candidates in each combination.

The Normal distribution had the lowest AIC in 40 region-by-group combinations (47.6%), followed by Gamma in 21 combinations (25.0%), Lognormal in 12 combinations (14.3%), Weibull4P in 10 combinations (11.9%) and the two-parameter Weibull in one combination (1.2%) (Table 4). The single-parameter Exponential distribution never had the lowest AIC and had ΔAIC ≥ 10 in all combinations. In 51 combinations (60.7%), two or more distributions had AIC values within 2 units of the smallest value, suggesting no clear preference between them. Weibull4P was adequate relative to the other candidates (ΔAIC < 10) across all 84 combinations, compared with Gamma in 74, Normal in 68, Lognormal in 66 and the two-parameter Weibull in 23 combinations (Figure 1 and Table 5). Weibull4P was also close to the lowest AIC (ΔAIC < 4) in 82 combinations. Its largest ΔAIC was 4.9, with a median of 2.8 and an interquartile range of 1.7–3.4. The largest ΔAIC values were higher for Normal (541.0), Gamma (131.9) and Lognormal (45.2).

**Figure 2:**
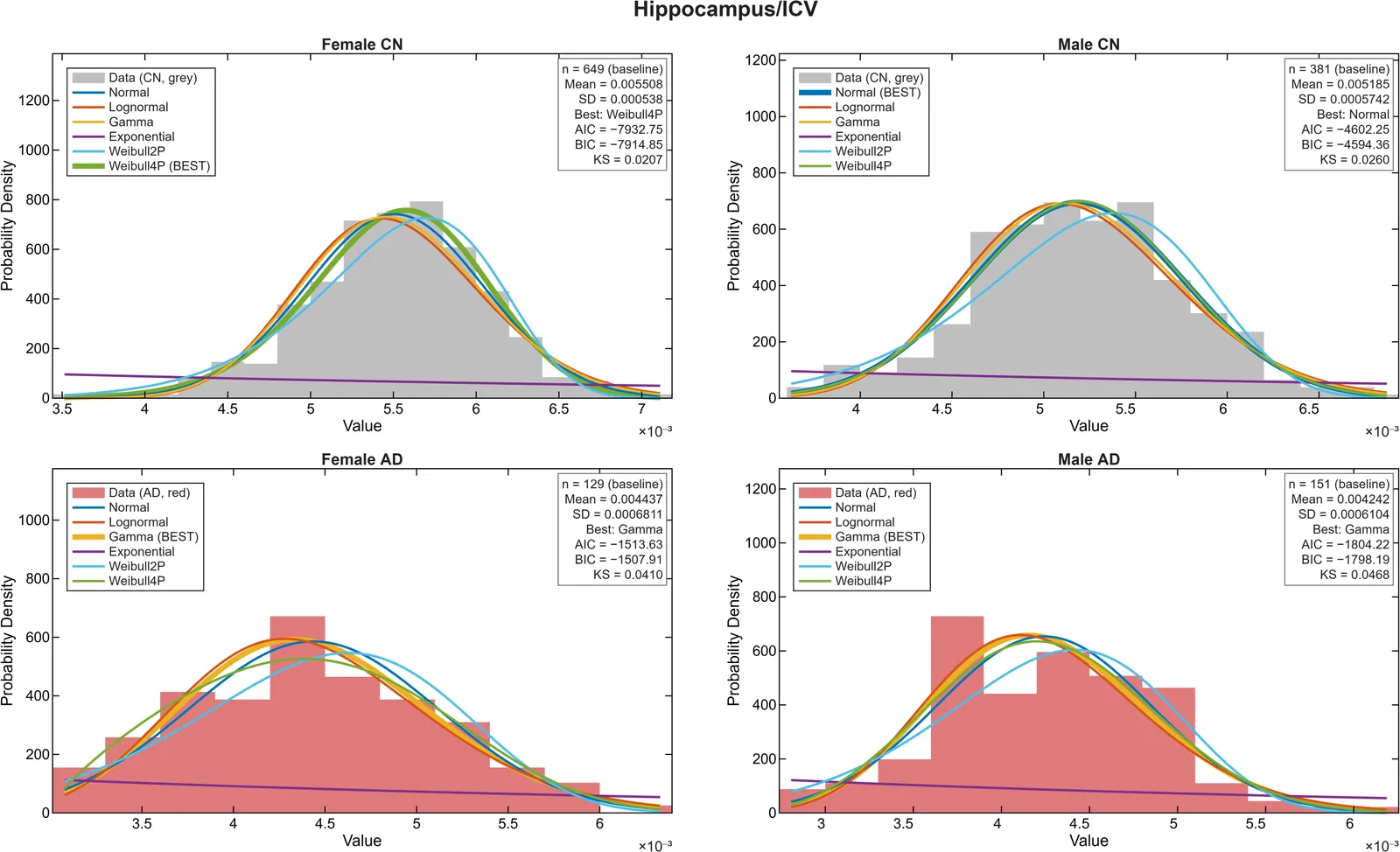
Fitted candidate distributions for ICV-normalized hippocampal volume by demographic and diagnostic group. Observed histograms are shown for cognitively normal (CN) participants and participants with Alzheimer’s disease (AD). The distribution with the lowest stability-qualified AIC in each group is shown as a thick line, and the remaining five candidate distributions are shown as thinner lines; each panel gives the sample size, mean, standard deviation, the lowest-AIC distribution and its AIC, BIC and KS distance. The corresponding fitted distributions for the remaining 20 brain regions are provided in Appendix A (Figure A1, Figure A2, Figure A3, Figure A4, Figure A5, Figure A6, Figure A7, Figure A8, Figure A9, Figure A10, Figure A11, Figure A12, Figure A13, Figure A14, Figure A15, Figure A16, Figure A17, Figure A18, Figure A19 and Figure A20).

**Figure 3:**
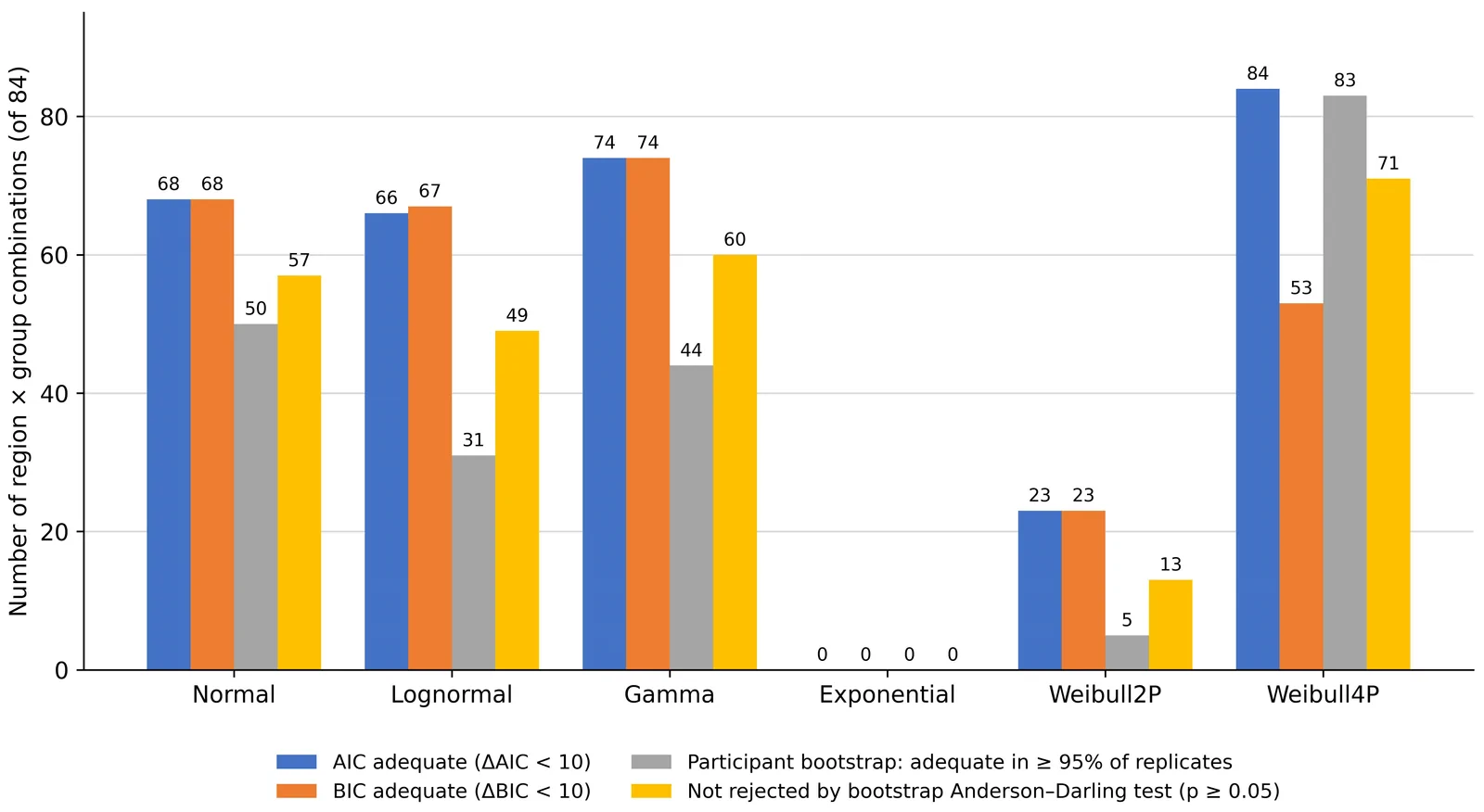
Number of the 84 region-by-group combinations in which each candidate distribution was adequate (difference from the lowest value < 10) under AIC and under BIC in the baseline analysis, and in at least 95% of the 500 participant-bootstrap replicates, and in which it was not rejected by the parametric-bootstrap Anderson–Darling test (*p* ≥ 0.05).

The distribution with the lowest AIC differed by region and demographic group (Table 4). The Normal distribution had the lowest AIC in 40 of the 72 tissue combinations and was adequate by the relative AIC in 67, while Weibull4P was adequate in all 72 and close to the lowest AIC in 70. In the 12 ventricular combinations, Weibull4P, Gamma and Lognormal each had the lowest AIC in four combinations. Normal was adequate by the relative AIC in only one, while Weibull4P was adequate and close to the lowest AIC in all 12. Figure 2 shows the fitted candidate distributions for ICV-normalized hippocampal volume in each of the four sex-by-diagnosis groups. The lowest-AIC distribution changed in 8 combinations when BIC was used. Normal had the lowest BIC most often (45 of 84 combinations, 53.6%), followed by Gamma (21, 25.0%), Lognormal (14, 16.7%), the two-parameter Weibull (2, 2.4%) and Weibull4P (2, 2.4%). AIC and BIC identified the same distribution in 76 of the 84 combinations (90.5%) (Figure 3). In all eight combinations where the criteria disagreed, AIC selected Weibull4P and BIC selected a two-parameter distribution. Weibull4P met the descriptive BIC support threshold (ΔBIC < 10) in 53 combinations, including 41 tissue and all 12 ventricular combinations. For all distributions, AICc gave the same relative adequacy counts as AIC. Weibull4P ranked first or second by KS distance in 78 combinations and by Cramér–von Mises distance in 76 combinations. In the Vuong test, none of the Normal, Lognormal and Gamma distributions fitted significantly better than Weibull4P in any combination and Weibull4P fitted significantly better than Normal in 9 of the 12 ventricular combinations. With the BIC correction, Normal was preferred in 47 of the 72 tissue combinations, whereas Weibull4P was preferred over Normal in 7 of the 12 ventricular combinations. All 84 baseline Weibull4P fits passed the StablePass criterion. All Hessians were positive definite, and no boundary solutions were identified; therefore, no Weibull4P fit was excluded from selection as the lowest-AIC distribution. Normality was rejected after FDR correction in 21 of the 84 combinations (25.0%), including 10 of the 12 ventricular combinations, and was not rejected in the remaining 63.

### 3.3. Absolute Goodness of Fit

All 252,000 refits were completed in the parametric-bootstrap tests. The Anderson–Darling test did not reject Weibull4P in 71 of the 84 combinations (*p* ≥ 0.05), the KS test did not reject it in 75 combinations, and the Cramér–von Mises test did not reject it in 74 combinations. None of the three tests rejected it in 69 combinations (Figure 3). The Normal distribution was not rejected in 57, 67 and 62 combinations, respectively; Gamma in 60, 65 and 64; Lognormal in 49, 58 and 54; and the two-parameter Weibull in 13, 25 and 17. Weibull4P was not rejected by the Anderson–Darling test in 11 of the 12 ventricular combinations; Normal was not rejected in one combination, and Gamma and Lognormal were not rejected in seven each. Weibull4P was rejected most often by the Anderson–Darling test in Female-CN, with six of the 21 combinations rejected. In seven of the 13 combinations where Weibull4P was rejected, the other five distributions were also rejected. In five combinations, Weibull4P was rejected while Normal was not rejected, and in 19 combinations, Normal was rejected while Weibull4P was not rejected. In 76 of the 84 combinations, the lowest-AIC distribution was not rejected by the uncorrected Anderson–Darling test. These tests measure the compatibility of each fitted distribution with the observed data, without comparing the candidates, and thus complement the relative AIC comparison reported in Section 3.2.

### 3.4. Participant-Level Stability, Cross-Validation and Age-Stratified Fits

The participant bootstrap indicated that the identity of the lowest-AIC distribution was uncertain in many combinations, whereas Weibull4P remained adequate in almost all replicates. Across the 84 combinations, the distribution with the lowest AIC in the baseline analysis also had the lowest AIC in a median of 54.7% of the 500 bootstrap replicates per combination (interquartile range, 43.4% to 67.0%; range 12.6% to 85.2%); it did so in more than half of the replicates in 52 combinations and it was the distribution that most frequently had the lowest AIC across the replicates in 70 of the 84 combinations (83.3%). In contrast, Weibull4P remained adequate (ΔAIC < 10) in at least 95% of the replicates in 83 of the 84 combinations, compared with 50 for the Normal, 44 for Gamma, 31 for Lognormal and 5 for the two-parameter Weibull distribution and it remained close (ΔAIC < 4) in at least 80% of the replicates in 82 combinations (Table 5 and Figure 3). The single exception was the parahippocampal gyrus in the Male-CN, in which Weibull4P was adequate in 94.2% of the replicates. These counts include Weibull4P refits that did not meet the StablePass criteria: across the 84 combinations, a median of 88.9% of the Weibull4P refits met this criterion (range, 57.8% to 100%) and Weibull4P was both adequate and stability-qualified in at least 95% of the replicates in 23 combinations.

In the cross-validation, Weibull4P was adequate out of sample in 55 of the 84 combinations; it was not adequate in the 23 combinations in which a held-out participant fell below the lower bound estimated from the training folds, and it was adequate in 55 of the remaining 61 (Supplementary Table S1). In the age-stratified refits, volume differed between the three age bands in 53 of the 84 combinations by the Kruskal–Wallis test after FDR correction. The same distribution had the lowest AIC in all three bands in only 10 combinations. In 203 of the 252 band fits, the two lowest-AIC distributions differed by less than 2 AIC units. Weibull4P was adequate by the relative AIC (ΔAIC < 10) in all three age bands in all 84 combinations and was close to the lowest AIC (ΔAIC < 4) in 246 of the 252 band fits. These counts include fits that did not meet the StablePass. Of the 252 Weibull4P bands, 54, across 41 combinations, did not meet StablePass and were therefore excluded from selection as the lowest-AIC distribution. Normal was adequate by the relative AIC in all three bands in 69 combinations, including 66 of the 72 tissue combinations and three of the 12 ventricular combinations.

### 3.5. Regional and Demographic Patterns: AD vs. CN and Female vs. Male

Before examining regional and demographic patterns in greater detail, we compared the volumes of cognitively normal (CN) and Alzheimer’s disease (AD) groups. This comparison was performed separately from the distribution-fitting analyses, using each participant’s baseline scan (Section 2.7). After age adjustment and FDR correction, 41 of the 42 sex-specific AD–CN comparisons were significant (q < 0.05); the exception was the anterior cingulate in females (q = 0.055). AD was associated with lower age-adjusted volume in all 36 tissue comparisons, covering the global gray and white matter measures and the medial temporal, temporal, parietal, frontal, and cingulate regions, and with larger volume in all 6 ventricular comparisons. The median absolute age adjusted difference was 9.4% of the CN mean (median standardized difference, 1.01) for the tissue regions and 37.5% (0.90) for the ventricles. The same 41 comparisons were significant when log-transformed volume was analyzed and the AD × age interaction was significant in 15 of the 42 comparisons, indicating that the AD–CN difference varied with age in some regions. Table 6 reports, for six regions of clinical interest, both the unadjusted percentage difference in mean volume, which is descriptive, and the age-adjusted difference with its 95% confidence interval. In 25 of the 42 comparisons, the *p*-value was smaller than 1 × 10^−15^. Among the six regions in Table 6, the largest unadjusted decreases were observed in the amygdala (−26.1% in females and −21.8% in males) and entorhinal cortex (−21.1% and −20.5%), followed by the hippocampus (−19.5% and −18.2%). Total gray matter showed smaller decreases (−7.7% and −6.6%). In contrast, the lateral and third ventricles showed larger volumes in the AD group (+53.1% and +38.2% for the lateral ventricles; +39.0% and +22.7% for the third ventricle). The largest relative differences overall were observed for the inferior lateral ventricle (+137.2% in females and +102.3% in males). The direction of the AD–CN difference was the same in females and males for every region. Beyond the mean, the two-sample KS test indicated that the CN and AD distributions differed in 41 of the 42 comparisons after FDR correction, and the bootstrap 95% confidence interval excluded no difference for the standard deviation in 11 comparisons and for the skewness in 8. Although several unadjusted percentage differences were numerically larger in females than in males, no sex × diagnosis interaction was tested, and these sex differences are therefore descriptive only.

## 4. Discussion

### 4.1. The Normal Distribution Described Most Tissue Regions but Not the Ventricles

The Normal distribution had the lowest AIC in 40 of 84 region-by-group combinations (47.6%), all of which were tissue combinations, and it was adequate in 67 of the 72 tissue combinations (Table 4 and Table 5). The Anderson–Darling test rejected normality in 21 of the 84 combinations after FDR correction, including 10 of the 12 ventricular combinations. These results indicate that, in this dataset, the Normal distribution provided a reasonable description of most tissue regions, whereas it did not describe the ventricular volumes, which were right-skewed in all 12 ventricular combinations (median skewness 0.92) and for which the Normal distribution was adequate in only one combination. The Anderson–Darling test assesses the absolute adequacy of a Normal distribution, and with 127 to 666 participants per combination, it can detect small departures from normality that may have limited practical importance [32]. In contrast, AIC compares the candidate models relative to each other, so a Normal distribution can have the lowest AIC among candidates even when normality is rejected, as occurred in 2 of the 40 combinations in which it had the lowest AIC. Normality should therefore be evaluated empirically rather than assumed in this type of data, particularly for the ventricular measures.

### 4.2. Weibull4P as an Adequate Description Across All Combinations

The central finding was that Weibull4P did not have the lowest AIC in most combinations, but it was the only candidate with ΔAIC < 10 in all 84 region-by-group combinations. It had the lowest AIC in 10 combinations, compared with 40 for Normal, and its largest ΔAIC was 4.9. Every other candidate had ΔAIC ≥ 10 in at least ten combinations. Weibull4P also remained within ΔAIC < 10 in at least 95% of participant-bootstrap replicates in 83 combinations and in all three age bands in every combination. These support counts include fits that did not meet StablePass. The Normal distribution was adequate by the AIC in 67 of the 72 tissue combinations but only one of the 12 ventricular combinations. Weibull4P was adequate in all 12 ventricular combinations and the Vuong test favored Weibull4P over Normal in nine of them. In the parametric-bootstrap Anderson–Darling tests, Weibull4P was not rejected in 71 combinations, compared with 60 for Gamma and 57 for Normal. These results support Weibull4P as a common description of tissue and ventricular volumes in the relative sense defined by the AIC. They do not establish it as the preferred distribution: its absolute fit was rejected in 13 combinations, and it met the descriptive support threshold under BIC in 53 combinations and cross-validation in 55. Thus, Normal is sufficient by the AIC criteria for most tissue combinations, while Weibull4P retains relative support across all regions and groups. The differences between the distributions become more apparent when the individual models are considered. The Normal and Lognormal distributions have more restricted shapes and do not provide the same flexibility for describing asymmetric distributions with different lower-tail behavior. The two-parameter Weibull provides greater flexibility through its shape parameter, but this parameter also influences the distribution’s skewness and tail behavior. These characteristics cannot be varied independently. The Exponentiated Weibull extends this framework by introducing an additional shape parameter and a location parameter [9,10]. These parameters provide greater flexibility in describing both the overall shape of the distribution and the position of its lower boundary. Such flexibility may be useful for regional brain volumes, which cannot be below zero and may show asymmetric distributions across different anatomical regions and diagnostic groups. In the present data, skewness was negative in 27 of the 84 combinations and positive in all 12 ventricular combinations; however, this same flexibility makes Weibull4P more difficult to estimate. All 84 Weibull4P fits met the stability criterion, but a small penalty was required in 15 combinations and the estimated location parameter was below zero in 44 fits, indicating that it did not represent a physical lower boundary for the observed brain volume measurements in those fits [16]. The findings may also reflect the biological heterogeneity associated with AD. Different patterns of disease accumulation have been observed in patients with clinical AD using in vivo tau-PET [33] and distinct regional atrophy patterns have been described in structural MRI [8]. Such heterogeneity, together with age-related volume loss that differs across brain structures [30], may contribute to variation in regional brain volumes that is not well represented by a symmetric distribution centered around a mean. However, the present analysis did not measure pathology, and the selected distribution cannot be attributed to a specific biological mechanism. A more flexible probability distribution may therefore provide a more appropriate statistical description of the observed variation without implying a particular underlying process. These findings align with a growing body of neuroimaging research examining alternatives to the Normal distribution. Some neuroimaging measures may be skewed or have heavier tails than a Gaussian model can adequately represent. For example, a methodological extension of a hierarchical Bayesian normative model used a flexible sinh-arcsinh distribution with neuroimaging data from 82 sites [7], motivated by the observation that the variability of many imaging phenotypes is skewed, kurtotic, or both. Large-scale normative brain charts have similarly modeled sex-specific, age-related changes in both the median and the variance of brain volumes using generalized additive models for location, scale, and shape [34,35]. In a study that derived hippocampal volume norms from cognitively normal ADNI participants, the Gamma distribution provided the best fit among the candidate distributions tested for FreeSurfer-derived hippocampal volume [36]. Although that study used a different modeling framework, its findings further support considering asymmetric distributions when characterizing hippocampal volume. Together, these findings and the results of the present study suggest that regional brain volumes may not always be adequately represented by a Normal distribution.

### 4.3. Model Selection Uncertainty and Stability of Adequacy

Several results indicate that the identity of the lowest-AIC distribution should be interpreted with caution in many individual combinations, which is why the study focused on adequacy. First, two or more distributions were within 2 AIC units of the lowest AIC in 51 of the 84 combinations; differences of this size are generally regarded as providing limited evidence for a clear preference between the models [20]. Second, in the participant bootstrap, the baseline lowest-AIC distribution had the lowest AIC in a median of only 54.7% of the replicates, and in 50% or fewer of the replicates in 32 combinations, although it was the distribution that most frequently had the lowest AIC in 70 of the 84 combinations. Third, in the age-stratified refits, the same distribution had the lowest AIC in all three age bands in only 10 combinations. Model-selection uncertainty of this kind can occur when several candidate models provide similar fits, and it is not reflected in estimates that are conditional on a single selected model [20]. BIC applies a stronger penalty for additional parameters because its penalty increases with sample size. In contrast, Weibull4P retained relative AIC support under each of these perturbations: it remained within 10 AIC units of the lowest AIC in at least 95% of the bootstrap replicates in 83 combinations and in all 252 age-band fits; these counts include fits that did not meet the StablePass criterion. Numerical stability was less consistent and StablePass was met in a median of 88.9% of the Weibull4P bootstrap refits (range, 57.8% to 100%), in at least 95% of the replicates in 23 combinations and in at least 80% in 57, and it was not met in 54 of the 252 age-band fits. In these data, a statement about which distributions are adequate is therefore more reproducible than a statement about which single distribution is best. The results also illustrate that the lowest-AIC distribution can depend on a small number of observations. In a preliminary analysis of the same data that did not include the ICV quality control (Section 2.2), Weibull4P had the lowest AIC in more combinations, because the few scans with implausible ICV values produced extreme ICV-normalized volumes that the four-parameter distribution could accommodate more readily than the two-parameter distributions; for cortical gray matter in the Male-AD group, for example, the log-likelihood advantage of Weibull4P over the Normal distribution decreased from 34.4 to 0.02 units after these scans were excluded.

### 4.4. AIC and BIC: Balancing Model Fit and Complexity

The differences between AIC and BIC observed in Section 3.2 illustrate that the two criteria place different emphasis on model fit and model complexity. AIC is designed to balance fit with model complexity and is commonly used when the objective is to minimize expected information loss or prediction error [20]. BIC applies a stronger penalty for additional parameters because its penalty increases with sample size. Consequently, the two criteria can favor different models when the improvement in fit from an additional parameter is relatively small. In the present study, AIC and BIC identified the same distribution in 76 of the 84 combinations (90.5%), and in all eight disagreements, AIC identified Weibull4P and BIC a two-parameter distribution. Under BIC, Weibull4P was adequate in 53 combinations, including all 12 ventricular combinations, but only 41 of the 72 tissue combinations. The improvement in fit provided by the two extra parameters of Weibull4P was therefore sufficient to keep it within the adequacy range under the fixed AIC penalty in every combination but not under the BIC penalty in 31 tissue combinations [18].

Neither criterion should therefore be considered the single “correct” criterion for the present analysis. Rather, they address somewhat different aspects of model selection [20]. If the primary objective is to describe the observed population while allowing additional flexibility when it improves fit, the AIC results support Weibull4P as adequate in every combination. If greater emphasis is placed on avoiding additional parameters unless strong evidence shows they are necessary, the BIC results provide greater support for the Normal, Gamma and Lognormal distributions in the tissue regions. Because the primary analysis used one baseline scan per participant, the sample sizes used in the AIC and BIC calculations correspond to the number of participants rather than the number of scans. For this reason, both AIC and BIC are reported throughout the study rather than selecting a single criterion as the definitive measure of model performance, and the conclusions of this study are framed in terms of the adequacy of each distribution and model-selection uncertainty rather than the identification of a single best distribution.

### 4.5. Regional and Demographic Patterns in Model Fit

The regional and demographic pattern of the lowest-AIC distributions showed some repeated patterns, although, given the selection uncertainty described in Section 4.3, individual cells of Table 4 should be interpreted with caution. Normal distribution had the lowest AIC in 8 to 12 of the 21 regions in each group and in all four groups for the fusiform gyrus and the superior parietal lobule, so that its selection did not differ markedly between diagnostic groups or sexes. The two-parameter Weibull had the lowest AIC in only one combination (Cortical white matter in the Female-AD group). Gamma or Lognormal had the lowest AIC in 25 tissue combinations; these combinations were mildly right-skewed (median skewness 0.32, compared with-0.02 in the combinations in which the Normal distribution had the lowest AIC) and the Normal distribution remained adequate in 21 of them. Weibull4P had the lowest AIC in 10 combinations, four of which were ventricular and five of which were Female-CN, the largest group (621 to 666 participants); a larger sample provides more information with which to support additional parameters [20], but this interpretation was not tested. The descriptive differences between females and males are relevant to the broader literature on sex differences in brain aging and AD-related brain atrophy. A large, multi-cohort study of 12,638 longitudinal scans from 4726 individuals across 14 cohorts [15] reported that men showed age-related decline in a greater number of brain regions than women, including during healthy aging, suggesting that sex-related differences in brain aging are not completely accounted for by AD diagnosis; an analysis of the smaller, single-cohort MIRIAD study reported sex differences in hippocampal atrophy progression [37]. This does not necessarily indicate a specific sex difference in AD-related atrophy. Rather, any such difference may be relatively small compared with other sources of variation or may depend on the characteristics of the cohort being studied. In the present analysis, the lowest-AIC distribution did not differ systematically between females and males. The Normal distribution had the lowest AIC in 8 and 10 of the 21 regions in the Female-CN and Male-CN groups, and in 12 and 10 in the Female-AD and Male-AD groups. Any such differences could reflect differences in age distribution (mean age at baseline, 69.3 vs. 72.1 years in the CN groups), cohort composition, or residual head-size effects, because proportional ICV normalization does not remove all size-related variation between the sexes [3,14]; it was not formally tested. Similarly, the direction of every AD–CN difference was the same in both sexes, and no sex × diagnosis interaction was tested, so the present results do not show that AD-related volume differences differ between females and males. The ventricular regions were different from those characterized primarily by tissue loss. In AD, regional brain tissue decreases [38], while ventricular volumes increase as surrounding tissue is lost [39]. All 12 ventricular combinations were right-skewed; normality was rejected in 10, and the Normal distribution was adequate by the AIC in only one. Weibull4P, Gamma and Lognormal each had the lowest AIC in four ventricular combinations. Weibull4P had the lowest AIC in all three ventricular regions in Female-CN, whereas Gamma or Lognormal had the lowest AIC in all six AD ventricular combinations. Weibull4P was close to the lowest AIC in all 12 ventricular combinations and the Vuong tests favored it over Normal in nine. For the ventricular volumes, a distribution that allows right skew should be used instead of assuming normality.

### 4.6. Statistical Implications

The parametric-bootstrap goodness-of-fit tests were used as an absolute check, in addition to AIC comparison. Distribution parameters were estimated from the same data being tested, so bootstrap calibration was used [26,27]. Weibull4P was not rejected by the Anderson–Darling test in 71 of the 84 combinations, more than any other candidate, and in 11 of the 12 ventricular combinations, where Normal was rejected in 11. Most Weibull4P rejections were seen in the larger groups, where smaller deviations from the fitted distribution can be detected by the test. A significant result means that there is evidence of a departure from the fitted distribution at the specified significance level, but this does not mean that it is inappropriate for every purpose. With 127 to 666 participants per combination, the size and nature of the departure should be considered alongside statistical significance [32]. Goodness of fit was evaluated for all six distributions in all 84 combinations and was not limited to the lowest-AIC distribution [40].

The distributional results also apply to statistical methods relying on the normality assumption. When regional brain volumes depart from normality, as the ventricular volumes did in the present data set, methods based on a Gaussian reference distribution may not capture the observed variation. This matters for reference range estimation, z-scores, parametric confidence intervals, or thresholds based on the mean and standard deviation. If there is a discrepancy, it would be most noticeable in the tails of the distribution, where the assumption of normality can have the most impact on recognizing unusually high or low measurements. This could be clinically relevant as brain volumes for different regions are often compared with normative values to look at unusual measurements or findings. The implications should be taken in conjunction with the model-selection uncertainty discussed in Section 4.3. Normative models assuming a single distribution selected for each region and group can understate uncertainty in the tails because the selected model was often close to its alternatives, and the model was not always stable when participants were resampled. A distribution that is adequate across all regions and groups, such as Weibull4P in the present data, may offer a common framework for such models. However, its estimated lower bound would need to be handled appropriately because new observations below it have zero density under the fitted model. The present analysis did not produce any normative percentile curves or z-scores from any of the fitted models, so the potential effect on normative thresholds is a topic for future investigation.

### 4.7. Limitations

First, the identity of the lowest-AIC distribution was not consistent in many combinations, and participant-bootstrap results should be viewed alongside the primary results. Second, Weibull4P was rejected by the parametric-bootstrap Anderson–Darling test in 13 of the 84 combinations. Its bootstrap refits used the penalty weight selected for the observed data and fewer starting points than the main analysis. The calibration was therefore conditional on the selected penalty weight rather than the full penalty-selection procedure. Weibull4P met the descriptive BIC threshold in 53 combinations and the cross-validation threshold in 55 (Supplementary Table S1). Held-out observations at or below its estimated lower bound have zero density under the fitted model and were assigned a finite density floor for scoring. The location estimate was also below zero in 44 fits and should not be interpreted as a physical minimum volume. Third, identifiability safeguards were required for Weibull4P. A small penalty was needed in 15 combinations, and the upper profile-likelihood confidence limit for the first shape parameter, a, was not reached in 17 combinations. This suggests limited precision for this parameter, although all baseline fits met StablePass. Numerical stability was less consistent during resampling: StablePass was achieved in a median of 88.9% of bootstrap refits and failed in 54 of the 252 age-band fits. AIC and BIC used the nominal parameter count and did not separately consider the penalty’s effect on effective model complexity. Fourth, data was collected across different ADNI phases and acquisition sites, with possible non-biological variability from scanners, acquisition protocols and processing procedures [41]. Model-selection sensitivity to phase or site was not explicitly assessed. The AD–CN comparisons were adjusted for age only, and the age-stratified refits used three broad age bands within each group. Regional sample sizes varied because of missing segmentations, whose effect on the fitted distributions was not formally assessed. Finally, expressing volumes as proportions of ICV does not remove all head-size-related variation. The results are specific to the analyzed ADNI sample, its age range and the six candidate distributions, and may not generalize to other datasets, processing pipelines or distribution families.

### 4.8. Future Directions

The present results support the potential value of studying the underlying distribution of MRI-derived regional measures, including candidate MRI-derived neuroimaging biomarkers, without assuming normality. This is especially important for normative modeling, where the reference distribution of a biomarker is used to describe the measurement of an individual compared to an appropriate population distribution [42]. Because of the asymmetric distributions, future normative modeling could be more flexible in terms of the shape of regional brain-volume distributions. The present study has outlined several directions that follow from the limitations and findings. First, models that recognize the longitudinal structure of the repeated observations, such as mixed-effects models or distributional regression models in which the location, scale, and shape parameters vary with age and other covariates [12,35], would enable the repeated scans to be used more effectively and would make it possible to formally investigate whether the shape of the distribution changes with age. Second, replication in an independent cohort and preferably also from different imaging sites and sensitivity analyses by ADNI phase or site would help to assess whether the observed distributional patterns are reproducible and represent biological variation or cohort- or scanner-specific effects. Third, future studies may investigate the association of the Weibull4P parameters with other biological markers of AD pathology, such as amyloid burden or tau burden. Multivariate analyses could also be performed to determine whether distributional characteristics are related across regions. Lastly, normative models based on Weibull4P with an appropriate treatment of its lower bound, uncertainty estimates for the cross-validated differences, and model-averaging approaches that do not depend on a single model selected by AIC and BIC [20] would be a useful extension of the present results.

## 5. Conclusions

In some statistical analyses of Alzheimer’s disease neuroimaging data, regional brain volumes are assumed to follow a Normal distribution, but this assumption is not routinely assessed. To examine this assumption, one baseline scan was used from each of 1343 ADNI participants after ICV quality control, and six candidate probability distributions were compared in each of 84 region-by-group combinations comprising 21 ICV-normalized brain regions and four sex-by-diagnosis groups. In this dataset, no single candidate distribution had the lowest AIC in every combination: the Normal distribution had the lowest AIC in 40 combinations (47.6%), followed by the Gamma (25.0%), Lognormal (14.3%), four-parameter Exponentiated Weibull (Weibull4P 11.9%) and two-parameter Weibull (1.2%) distributions, and the Exponential distribution in none. Two or more distributions were within 2 AIC units of each other in 51 combinations, and the lowest-AIC distribution was reproduced in a median of 54.7% of the participant-bootstrap replicates. Weibull4P was the only distribution that was adequate (ΔAIC < 10) in all 84 combinations, and it remained adequate in at least 95% of the bootstrap replicates in 83 combinations and in all three age bands in every combination; in the Vuong tests, the Normal, Lognormal and Gamma distributions never fitted significantly better than Weibull4P. In the parametric-bootstrap goodness-of-fit tests, Weibull4P was not rejected by the Anderson–Darling test in 71 of 84 combinations, more than any other candidate distribution, and in 11 of the 12 ventricular combinations. The Normal distribution described most tissue regions well but not the ventricular volumes, in which normality was rejected in 10 of 12 combinations. The additional parameters of Weibull4P were not always required: it was adequate under BIC in 53 combinations, and in the cross-validation in 55, mainly because of its estimated lower bound. The overall pattern of medial temporal lobe atrophy and ventricular enlargement was consistent with the established features of AD; 41 of the 42 sex-specific AD–CN comparisons were significant after age adjustment and FDR correction. These results indicate that, in this ADNI sample and under the six-candidate framework, the Normal distribution is sufficient for most tissue regions, whereas the Exponentiated Weibull distribution is the only candidate that remained adequate relative to the other candidates in every region-by-group combination, including the ventricular regions, at the cost of two additional parameters and weaker out-of-sample performance. Future studies should address these distributional features explicitly in the tails of the distributions, where diagnostic decisions and normative percentile thresholds are made and should consider model selection uncertainty when regional brain volume measurements are used for clinical decision-making.

## Figures and Tables

**Table 1:** The six candidate probability distributions used in the six-model comparison, their parameters, and estimation method. The number before each parameter list is the number of freely estimated parameters (k) used in AIC and BIC calculations.

Distribution	Parameters	Estimation Method
Normal	2 (µ, σ)	fitdist
Lognormal	2 (µ log, σ log)	fitdist
Gamma	2 (shape a, scale b)	fitdist
Exponential	1 (mean µ)	fitdist
Weibull (2-parameter)	2 (scale A, shape B)	fitdist
Exponentiated Weibull (4-parameter)	4 (shape a, scale b, shape c, location loc)	fminunc (multistart, ridge-stabilized)

**Table 2:** Probability density and cumulative distribution functions for the six candidate distributions in Table 1.

Distributions	Probability Density Function	Cumulative Distribution Function
Normal	f(x) = (1/(σ√(2π)))·exp(−(x − μ)^2^/(2σ^2^))	F(x) = 0.5[1 + erf((x − μ)/(σ√2))]
Lognormal	f(x) = (1/(xσ√(2π)))·exp(−(ln(x) − μ)^2^/(2σ^2^)), x > 0	F(x) = 0.5[1 + erf((ln(x) − μ)/(σ√2))]
Gamma	f(x) = x^(a − 1)·exp(−x/b)/[Γ(a)·b^a], x > 0	F(x) = γ (a, x/b)/Γ(a)
Exponential	f(x) = (1/μ)·exp(−x/μ), x ≥ 0	F(x) = 1 − exp(−x/μ)
Weibull (2-parameter)	f(x) = (B/A)·(x/A)^(B − 1)·exp(−(x/A)^B), x ≥ 0	F(x) = 1 − exp(−(x/A)^B)
Exponentiated Weibull (4-parameter)	f(x) = (ac/b)·z^(c − 1)·exp(−z^c)·[1−exp(−z^c)]^(a − 1), z = (x − loc)/b, x > loc	F(x) = [1 − exp(−z^c)]^a, z = (x − loc)/b, x > loc

**Table 3:** Sample composition by demographic group. Participant counts and baseline ages correspond to the baseline (one scan per participant) framework used in the primary analysis; scan counts include all the repeated scans.

Group	Scans (n)	Unique Participants	Mean Age (SD), Years: Baseline/All Scans
Female-CN	1592	666	69.3(7.4)/72.8(7.5)
Male-CN	1144	389	72.1(7.4)/75.1(7.4)
Female-AD	294	134	73.0(8.0)/73.4(8.0)
Male-AD	379	154	74.8(8.1)/75.1(8.1)
All CN	2736	1055	70.3(7.5)/73.7(7.5)
All AD	673	288	74.0(8.1)/74.4(8.1)
Overall	3409	1343	71.1(7.8)/73.9(7.7)

**Table 4:** Distribution of the lowest stability-qualified AIC for each of the 21 ICV-normalized regions across the four demographic groups (baseline data). The value in parentheses is the AIC difference in Weibull4P from the lowest AIC (ΔAIC); Weibull4P was adequate (ΔAIC < 10) in every combination. Cells without a value are those in which Weibull4P had the lowest AIC.

Region	Female-CN	Male-CN	Female-AD	Male-AD
Amygdala/ICV	Normal (2.5)	Normal (1.0)	Gamma (3.8)	Lognormal (2.4)
Anterior Cingulate/ICV	Weibull4P	Gamma (2.6)	Normal (2.1)	Gamma (3.1)
Cortical GM/ICV	Normal (1.2)	Weibull4P	Normal (0.2)	Normal (4.0)
Cortical WM/ICV	Lognormal (2.8)	Normal (2.6)	Weibull2P (2.2)	Normal (3.0)
Fusiform/ICV	Normal (2.4)	Normal (2.9)	Normal (1.9)	Normal (3.7)
Hippocampus/ICV	Weibull4P	Normal (3.8)	Gamma (0.5)	Gamma (3.4)
Inferior Lateral Ventricle/ICV	Weibull4P	Weibull4P	Lognormal (1.1)	Gamma (2.1)
Inferior Parietal/ICV	Gamma (2.3)	Gamma (2.8)	Normal (3.5)	Normal (3.0)
Lateral Ventricle/ICV	Weibull4P	Lognormal (1.1)	Gamma (3.3)	Gamma (3.2)
Middle Temporal/ICV	Normal (1.9)	Lognormal (3.0)	Normal (3.1)	Lognormal (3.2)
Parahippocampal/ICV	Gamma (3.7)	Lognormal (2.7)	Normal (3.5)	Gamma (3.3)
Posterior Cingulate/ICV	Normal (2.4)	Normal (2.8)	Weibull4P	Normal (3.9)
Precuneus/ICV	Gamma (3.6)	Normal (2.3)	Gamma (3.9)	Normal (3.0)
Rostral Middle Frontal/ICV	Lognormal (2.6)	Lognormal (4.9)	Normal (3.8)	Lognormal (3.9)
Superior Frontal/ICV	Gamma (3.9)	Normal (3.0)	Normal (1.5)	Normal (3.4)
Superior Parietal/ICV	Normal (3.2)	Normal (2.9)	Normal (3.9)	Normal (2.0)
Superior Temporal/ICV	Normal (3.9)	Normal (2.9)	Gamma (2.1)	Normal (3.4)
Third Ventricle/ICV	Weibull4P	Lognormal (0.4)	Lognormal (2.5)	Gamma (1.8)
Total GM/ICV	Normal (0.5)	Weibull4P	Normal (0.4)	Gamma (3.7)
Total Entorhinal/ICV	Gamma (1.1)	Gamma (1.9)	Normal (3.3)	Weibull4P
Total Inferior Temporal/ICV	Gamma (3.3)	Normal (3.4)	Normal (3.2)	Normal (4.1)

**Table 5:** Number of the 84 region-by-group combinations in which each candidate distribution had the lowest value and was adequate (difference from the lowest value < 10) under AIC and BIC, was most frequently the lowest-AIC distribution and was adequate in at least 95% of the 500 participant-bootstrap replicates.

Distribution	AIC: Lowest/Adequate	BIC: Lowest/Adequate	Baseline Participant Bootstrap, Most Frequent/Adequate in ≥95%
Normal	40/68	45/68	43/50
Lognormal	12/66	14/67	14/31
Gamma	21/74	21/74	14/44
Exponential	0/0	0/0	0/0
Weibull2P	1/23	2/23	4/5
Weibull4P	10/84	2/53	9/83
Agreement with stability-qualified AIC	—	76/84 (90.5%)	70/84 (83.3%)

**Table 6:** AD–CN differences in ICV-normalized volume by sex, with age-adjusted estimates. Other results for all 42 comparisons are given in Supplementary Table S1.

Region	Sex	N (CN/AD)	% Difference (Unadjusted)	Age-Adjusted Difference, ×10^−3^ (95% CI)	FDR q
Amygdala/ICV	Female	649/128	−26.1%	−0.527 (−0.591, −0.463)	<1 × 10^−15^
	Male	380/151	−21.8%	−0.429 (−0.491, −0.367)	<1 × 10^−15^
Hippocampus/ICV	Female	649/129	−19.5%	−0.969 (−1.091, −0.847)	<1 × 10^−15^
	Male	381/151	−18.2%	−0.869 (−0.981, −0.758)	<1 × 10^−15^
Total Entorhinal/ICV	Female	649/128	−21.1%	−0.525 (−0.610, −0.440)	<1 × 10^−15^
	Male	380/151	−20.5%	−0.500 (−0.593, −0.406)	<1 × 10^−15^
Total GM/ICV	Female	666/133	−7.7%	−28.379 (−33.107, −23.650)	<1 × 10^−15^
	Male	388/154	−6.6%	−23.695 (−28.406, −18.983)	<1 × 10^−15^
Lateral Ventricle/ICV	Female	666/132	+53.1%	7.892 (5.838, 9.945)	1.9 × 10^−13^
	Male	388/154	+38.2%	7.526 (5.565, 9.486)	2.9 × 10^−13^
Third Ventricle/ICV	Female	666/132	+39.0%	0.295 (0.220, 0.370)	4.5 × 10^−14^
	Male	388/154	+22.7%	0.211 (0.144, 0.278)	1.6 × 10^−9^

**Figure A1:**
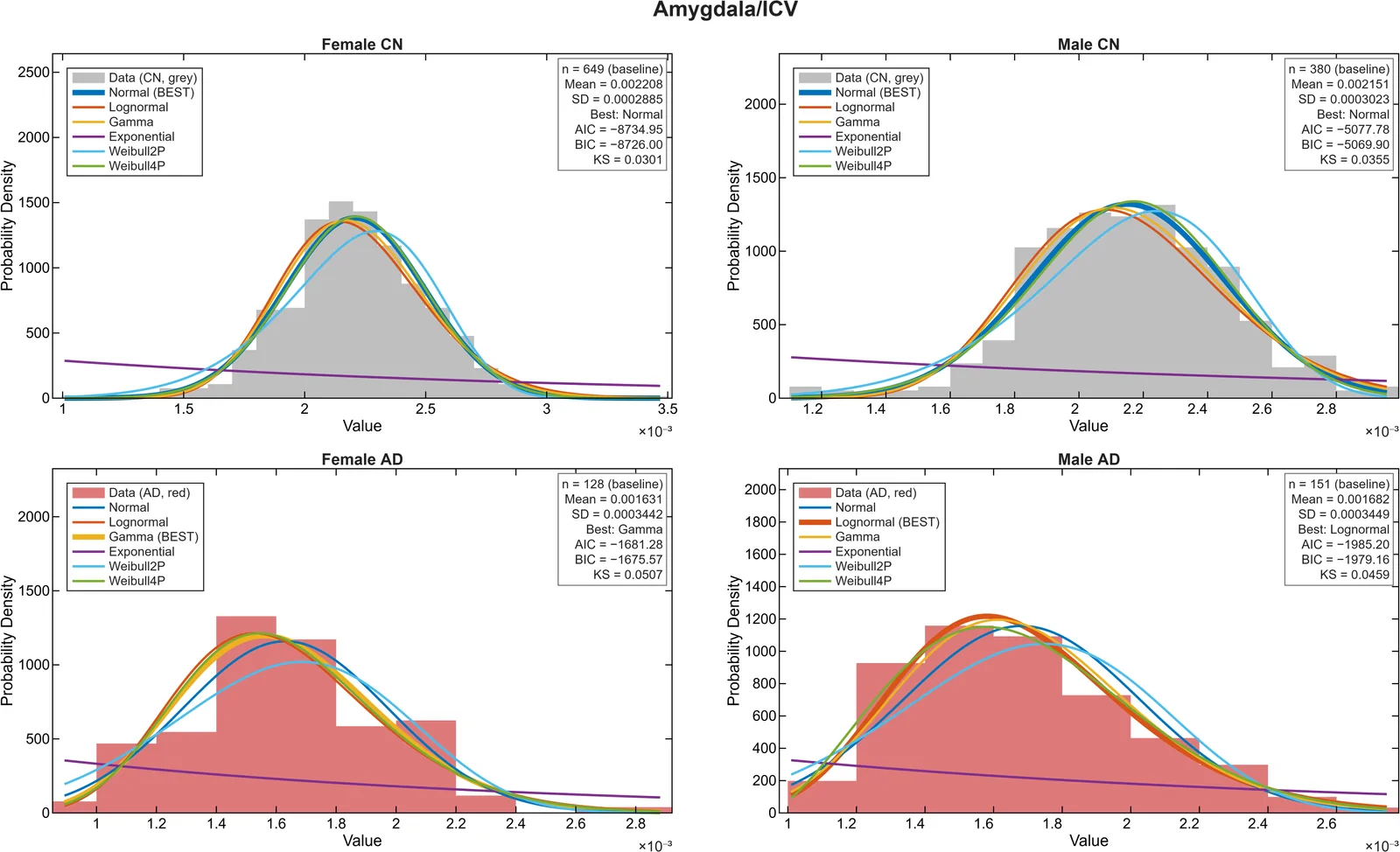
Amygdala volume/ICV—lowest AIC: Normal in Female-CN and Male-CN; Gamma in Female-AD; Lognormal in Male-AD. Weibull4P was adequate in all four groups (largest ΔAIC, 3.8).

**Figure A2:**
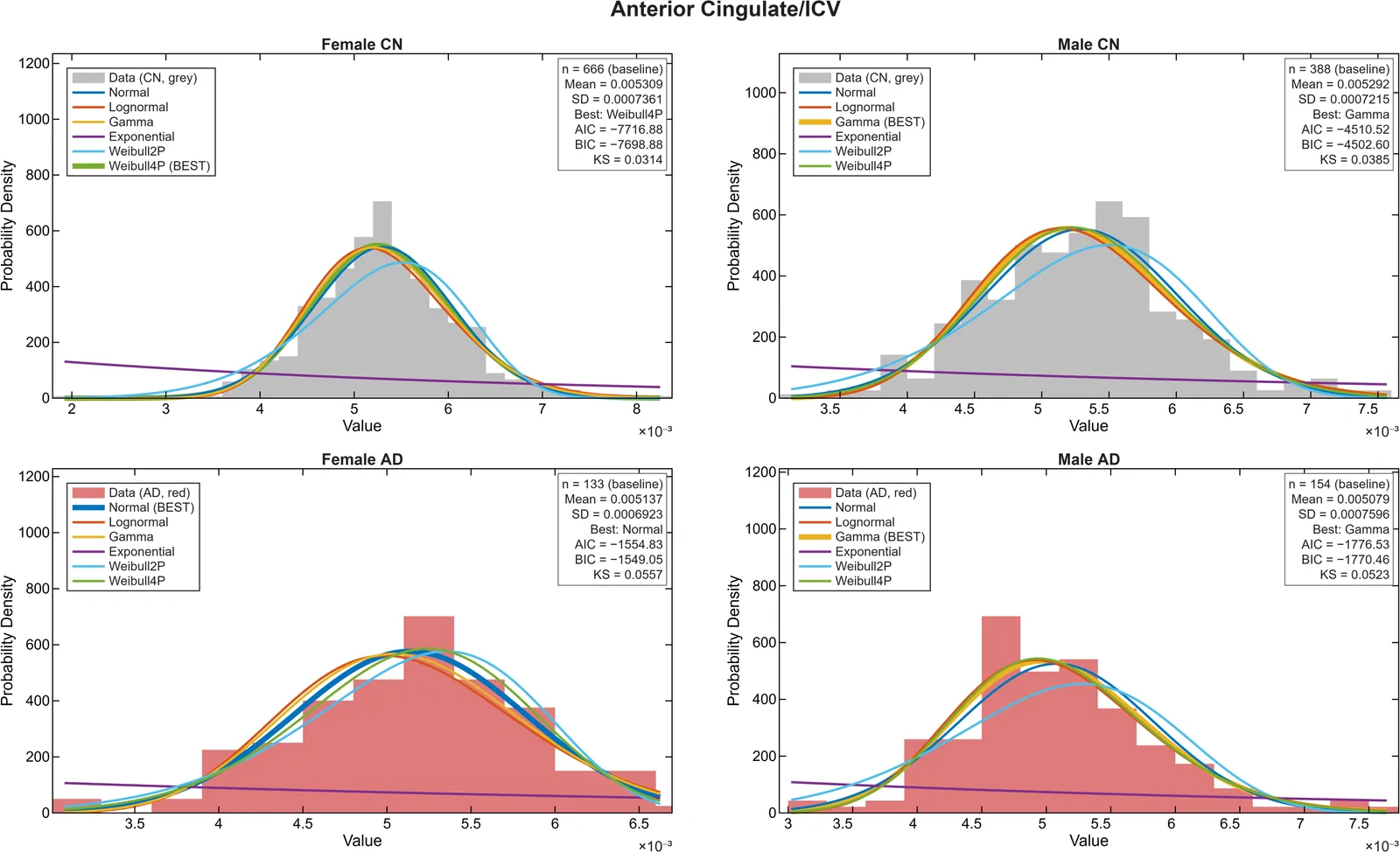
Anterior cingulate cortex volume/ICV—lowest AIC: Weibull4P in Female-CN; Gamma in Male-CN and Male-AD; Normal in Female-AD. Weibull4P was adequate in all four groups (largest ΔAIC, 3.1).

**Figure A3:**
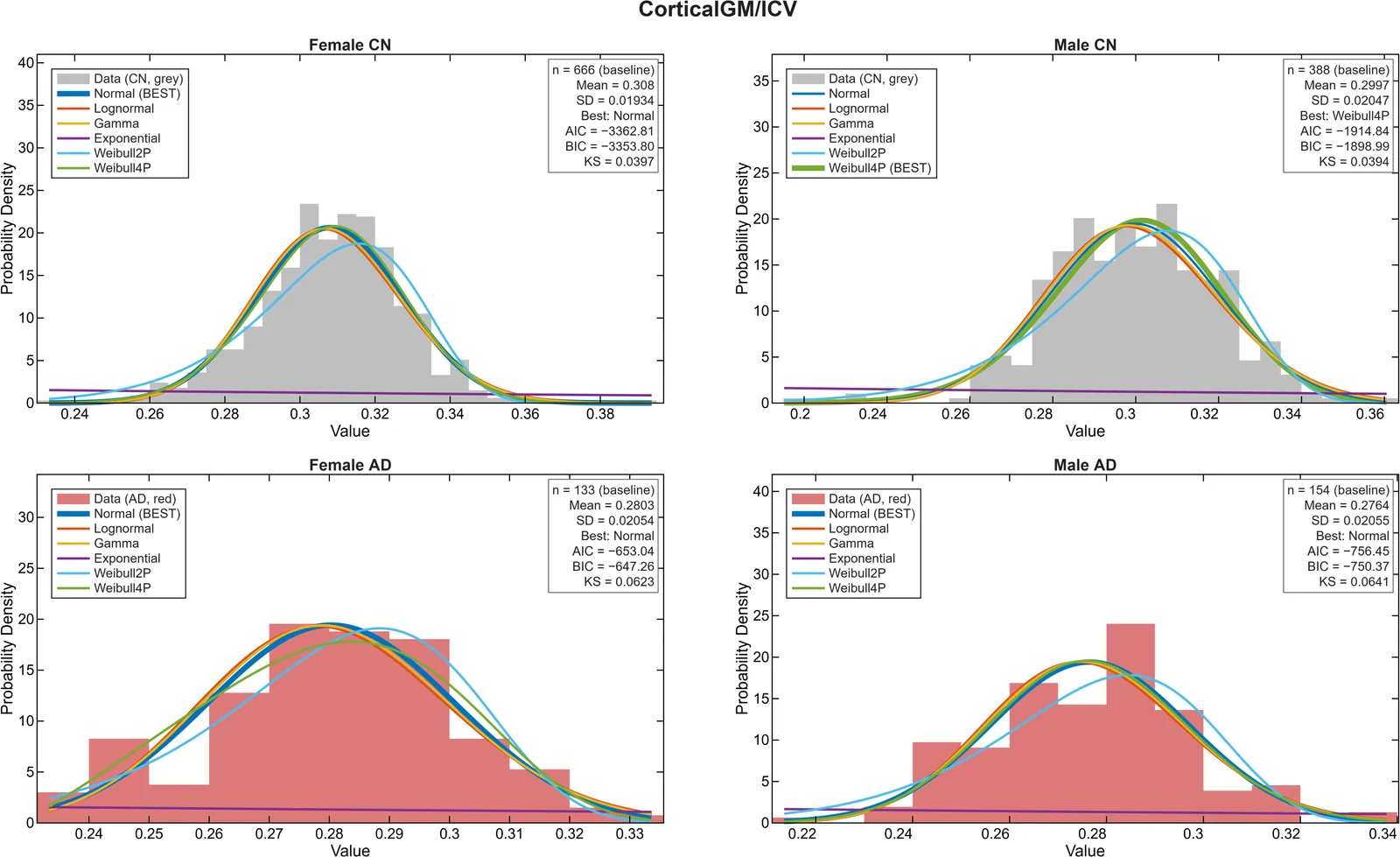
Cortical gray matter volume/ICV—lowest AIC: Normal in Female-CN, Female-AD, and Male-AD; Weibull4P in Male-CN. Weibull4P was adequate in all four groups (largest ΔAIC, 4.0).

**Figure A4:**
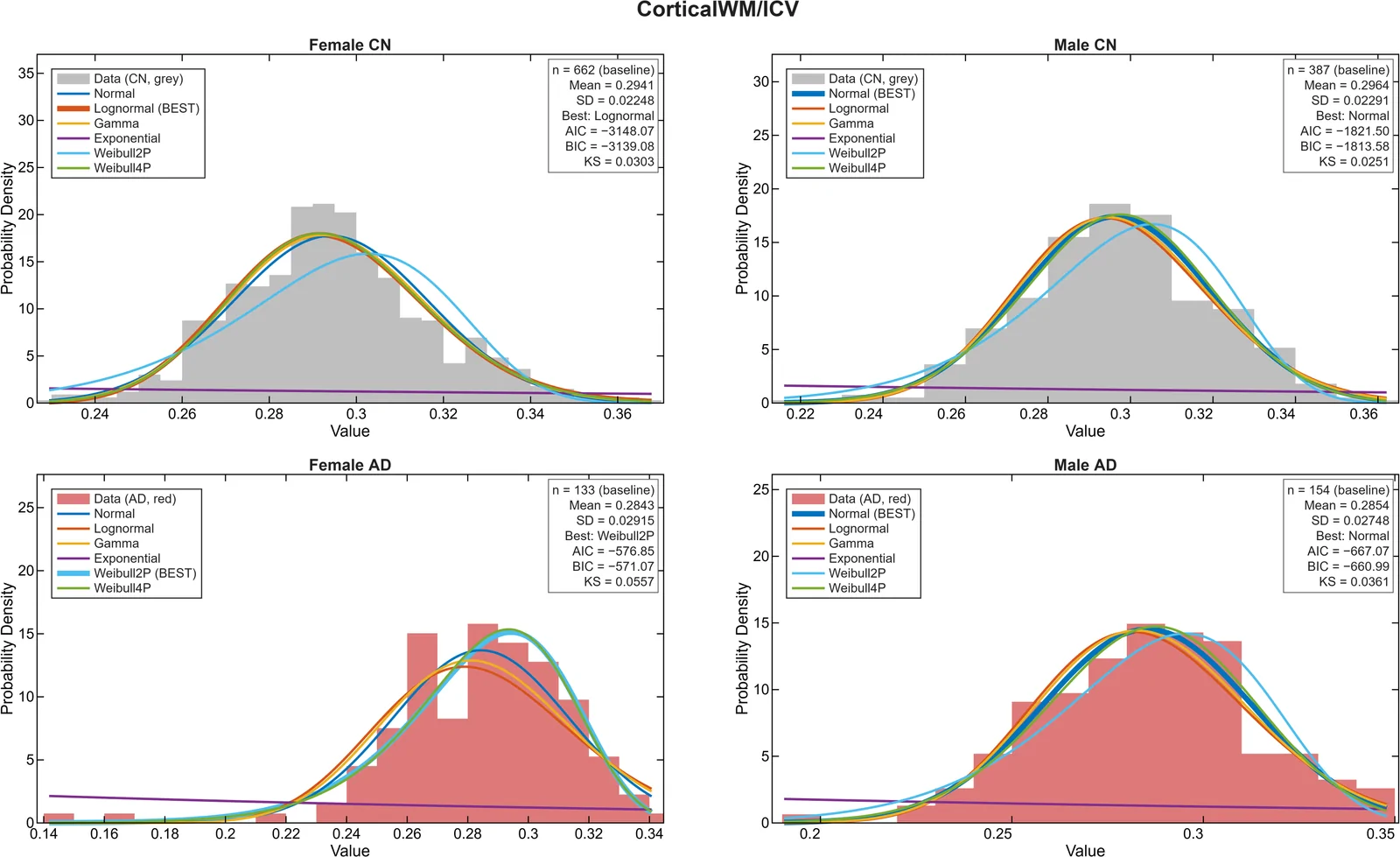
Cortical white matter volume/ICV—lowest AIC: Lognormal in Female-CN; Normal in Male-CN and Male-AD; Weibull2P in Female-AD. Weibull4P was adequate in all four groups (largest ΔAIC, 3.0).

**Figure A5:**
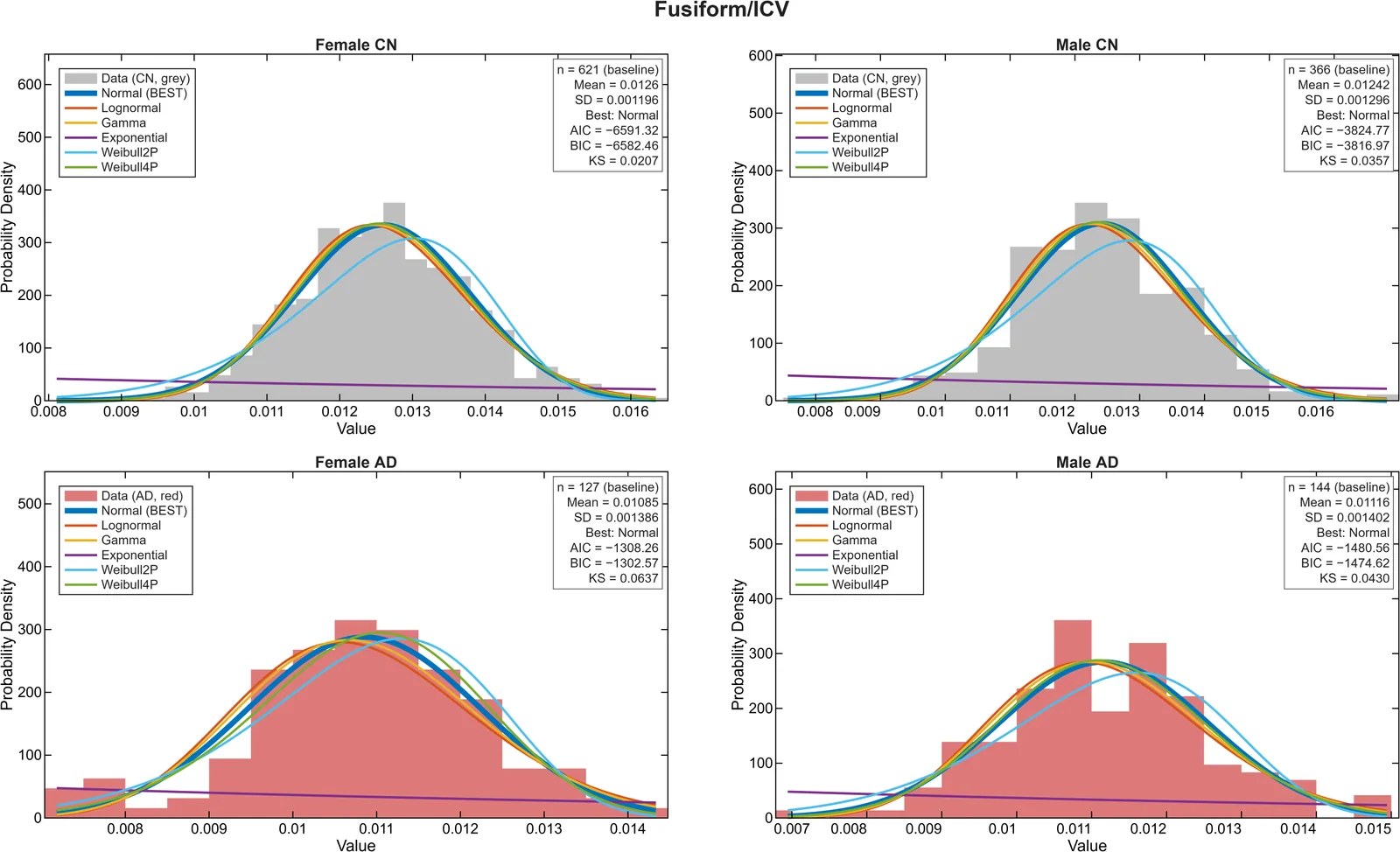
Fusiform gyrus volume/ICV—Normal had the lowest AIC in all four groups. Weibull4P was adequate in all four groups (largest ΔAIC, 3.7).

**Figure A6:**
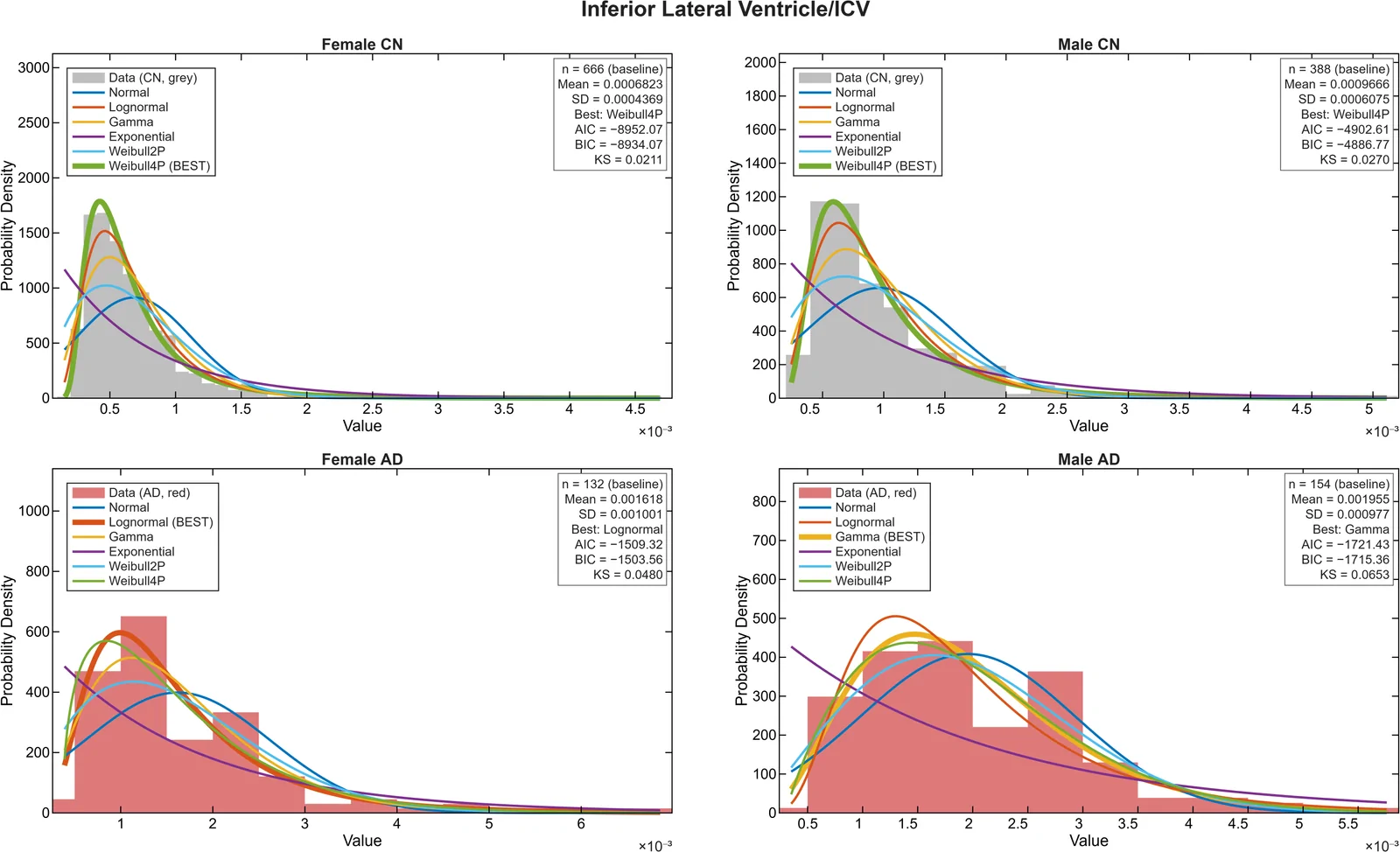
Inferior lateral ventricle volume/ICV—lowest AIC: Weibull4P in Female-CN and Male-CN; Lognormal in Female-AD; Gamma in Male-AD. Weibull4P was adequate in all four groups (largest ΔAIC, 2.1).

**Figure A7:**
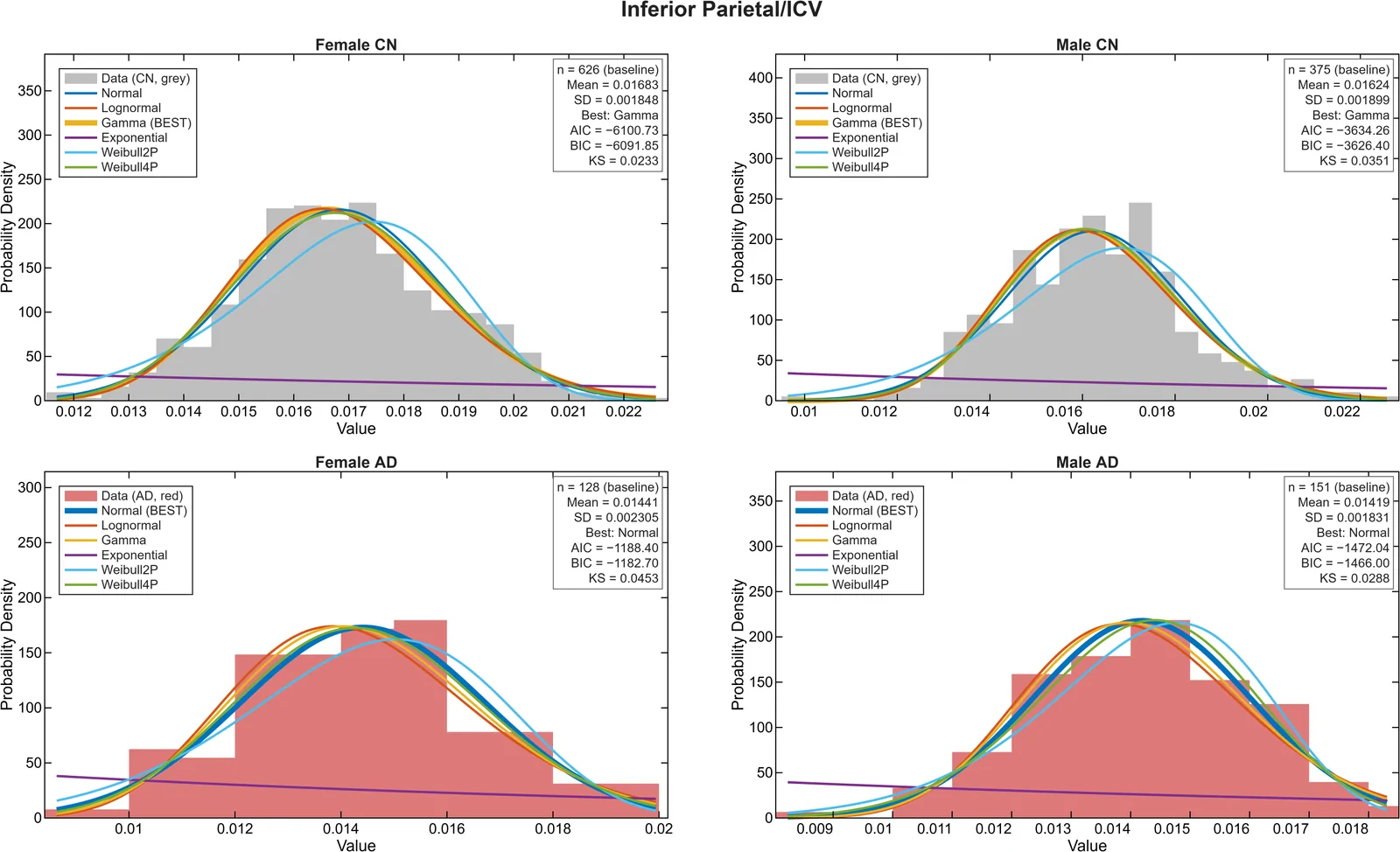
Inferior parietal lobule volume/ICV—lowest AIC: Gamma in Female-CN and Male-CN; Normal in Female-AD and Male-AD. Weibull4P was adequate in all four groups (largest ΔAIC, 3.5).

**Figure A8:**
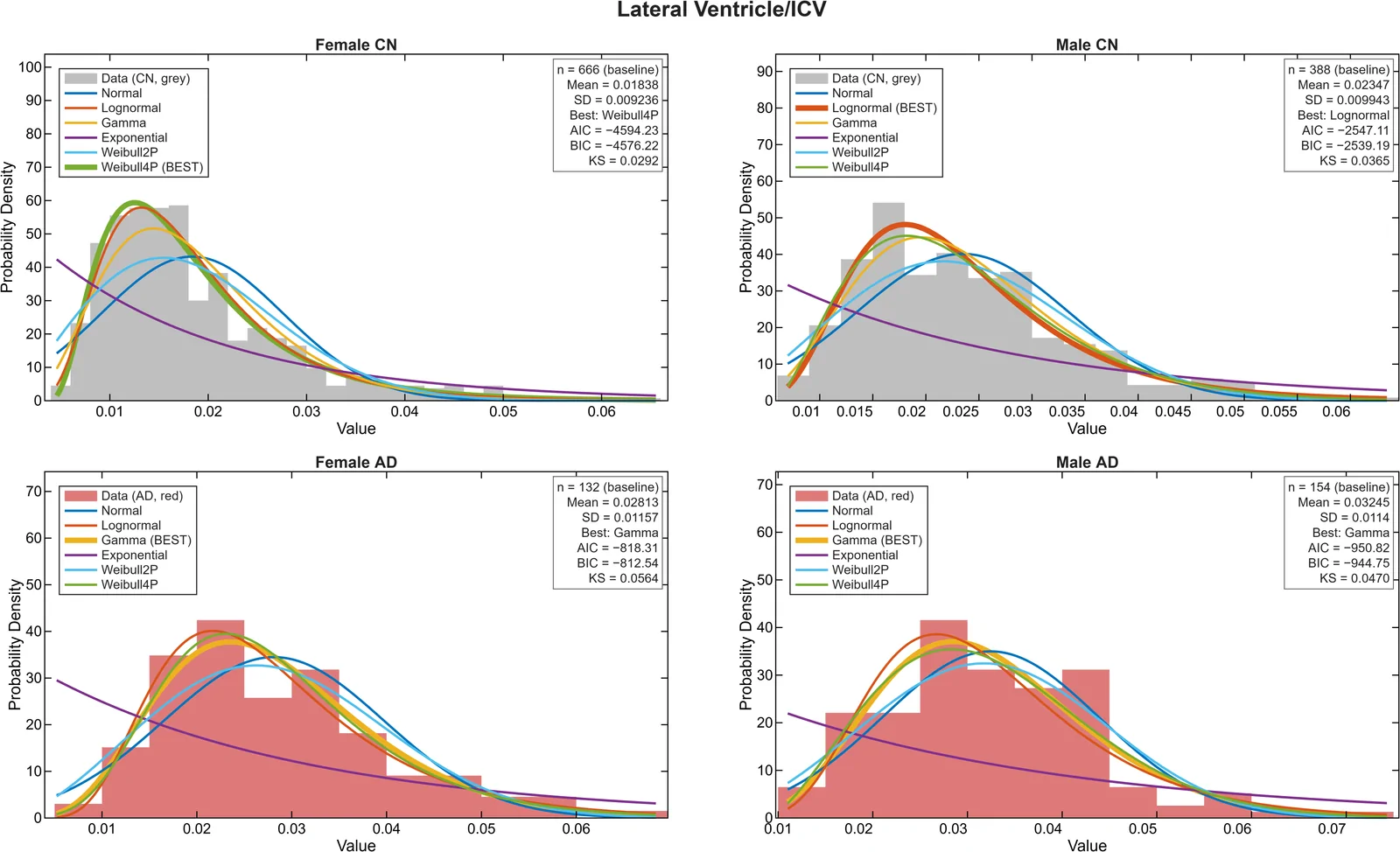
Lateral ventricle volume/ICV—lowest AIC: Weibull4P in Female-CN; Lognormal in Male-CN; Gamma in Female-AD and Male-AD. Weibull4P was adequate in all four groups (largest ΔAIC, 3.3).

**Figure A9:**
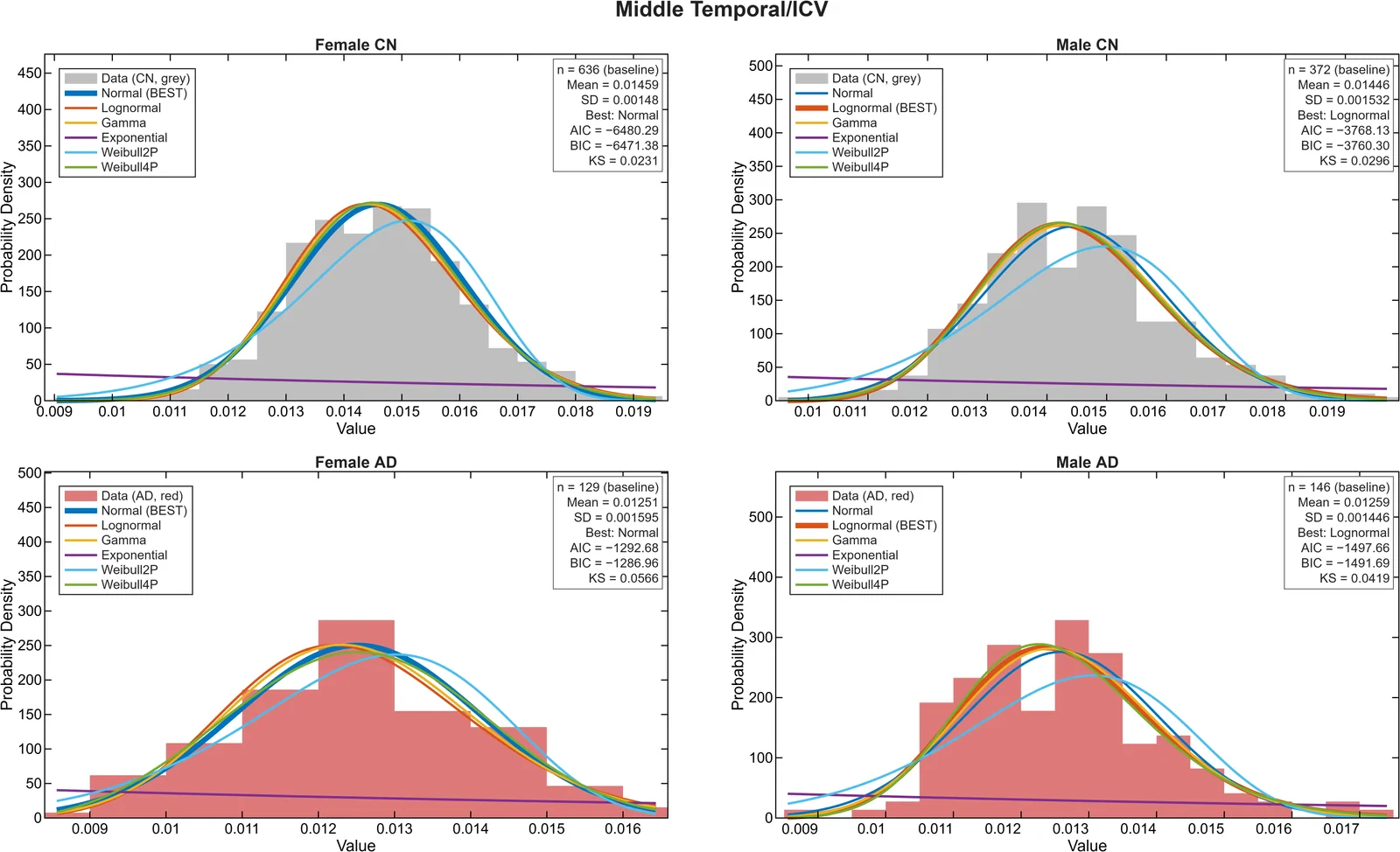
Middle temporal gyrus volume/ICV—lowest AIC: Normal in Female-CN and Female-AD; Lognormal in Male-CN and Male-AD. Weibull4P was adequate in all four groups (largest ΔAIC, 3.2).

**Figure A10:**
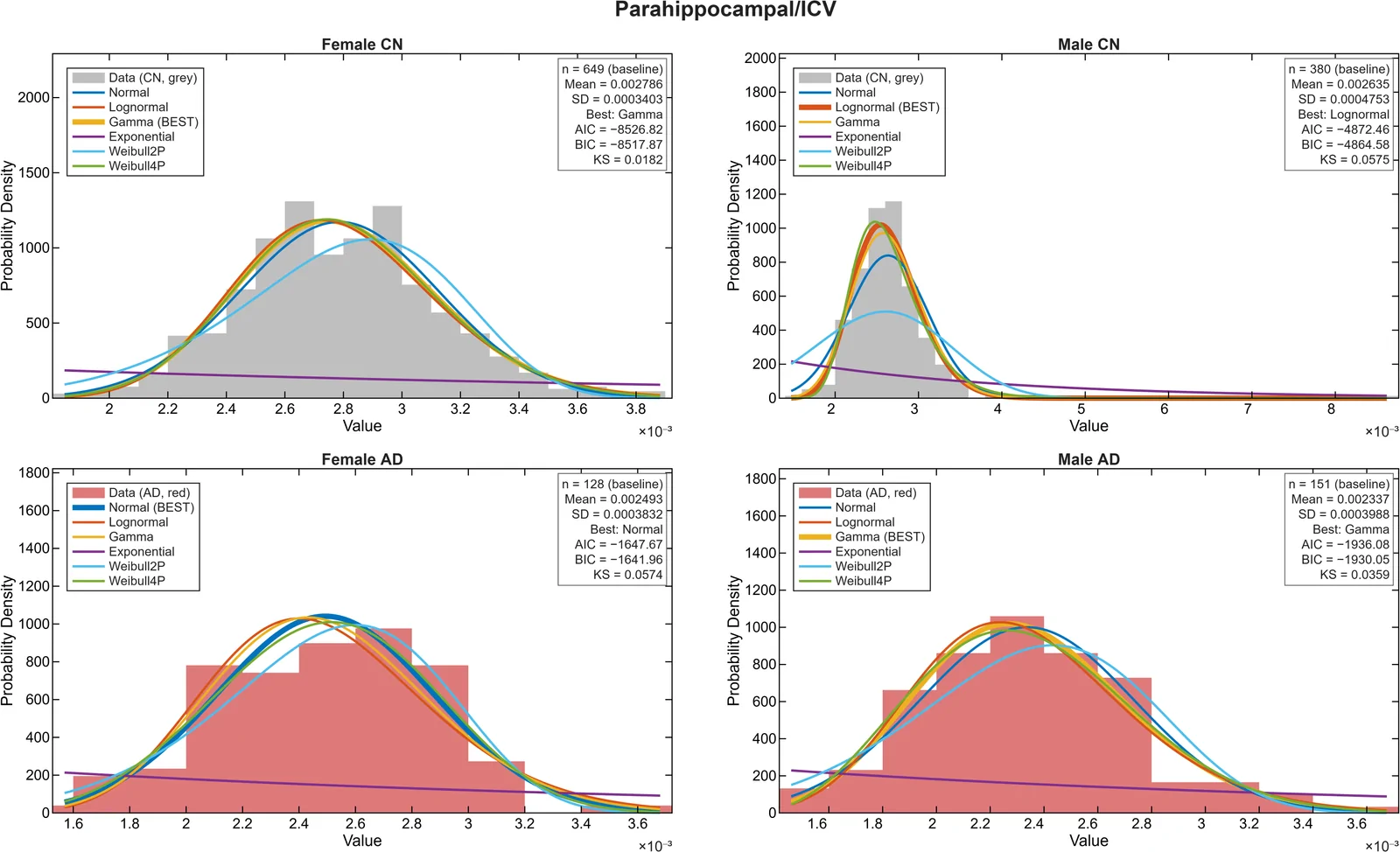
Parahippocampal gyrus volume/ICV—lowest AIC: Gamma in Female-CN and Male-AD; Lognormal in Male-CN; Normal in Female-AD. Weibull4P was adequate in all four groups (largest ΔAIC, 3.7).

**Figure A11:**
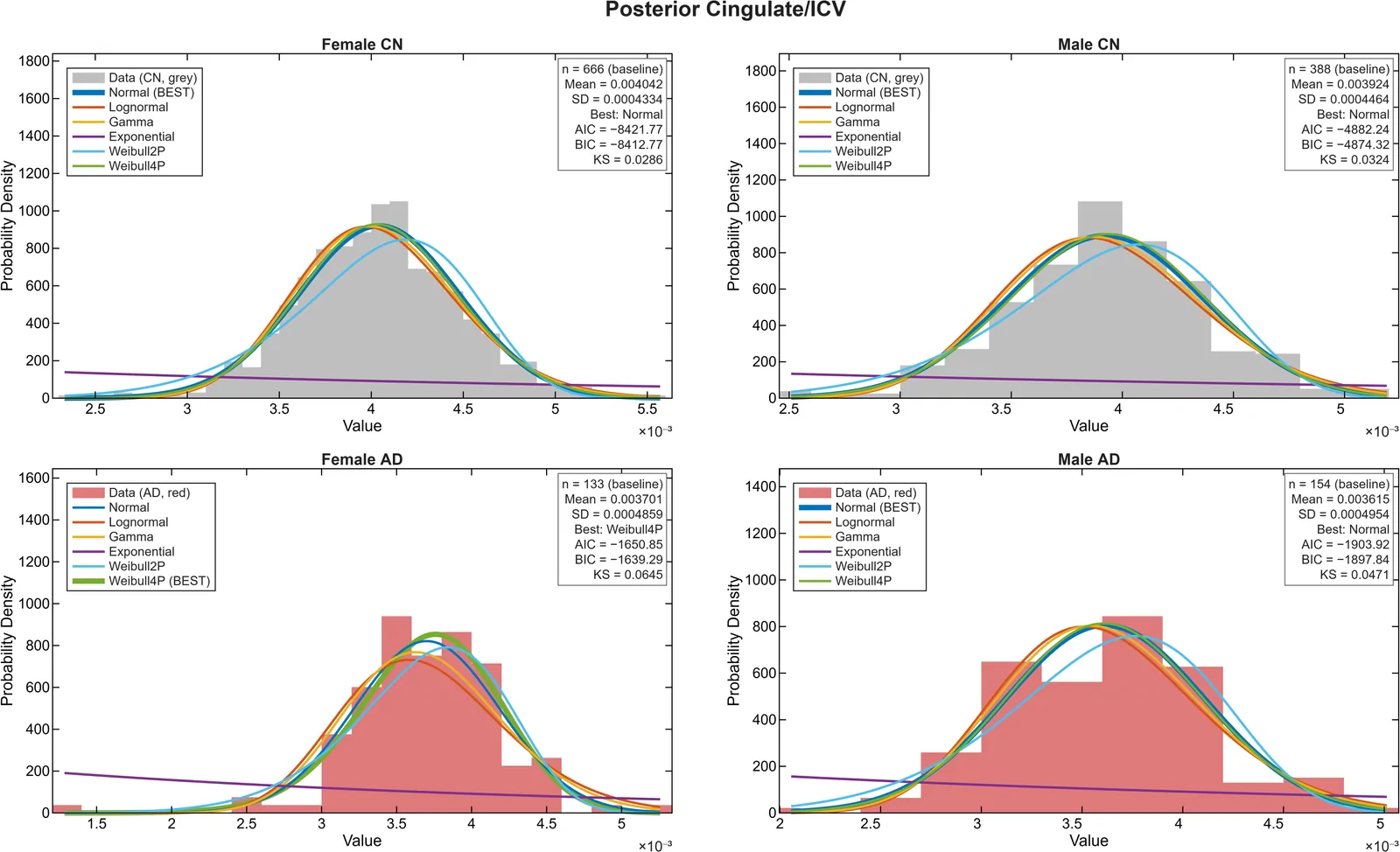
Posterior cingulate cortex volume/ICV—lowest AIC: Normal in Female-CN, Male-CN, and Male-AD; Weibull4P in Female-AD. Weibull4P was adequate in all four groups (largest ΔAIC, 3.9).

**Figure A12:**
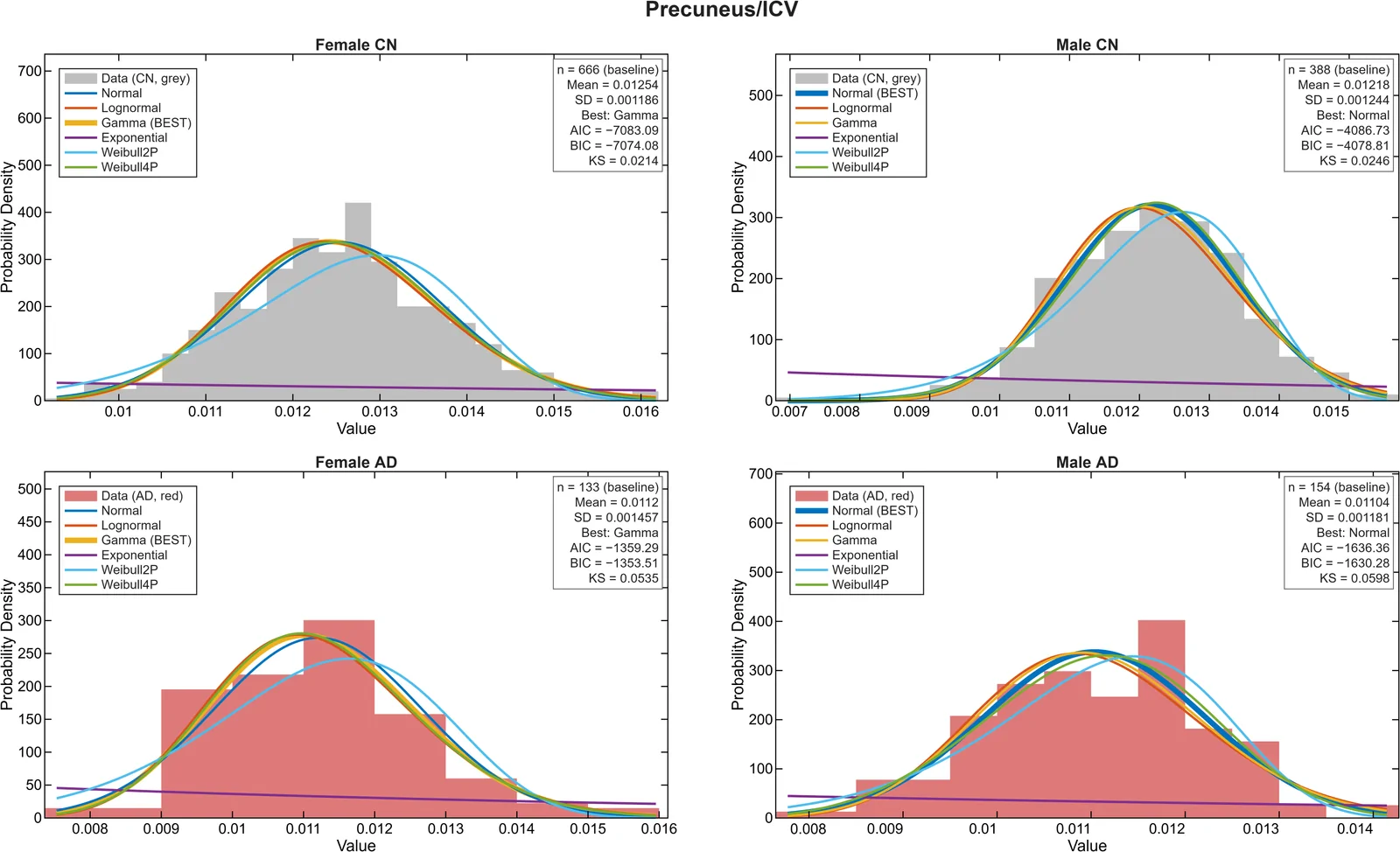
Precuneus volume/ICV—lowest AIC: Gamma in Female-CN and Female-AD; Normal in Male-CN and Male-AD. Weibull4P was adequate in all four groups (largest ΔAIC, 3.9).

**Figure A13:**
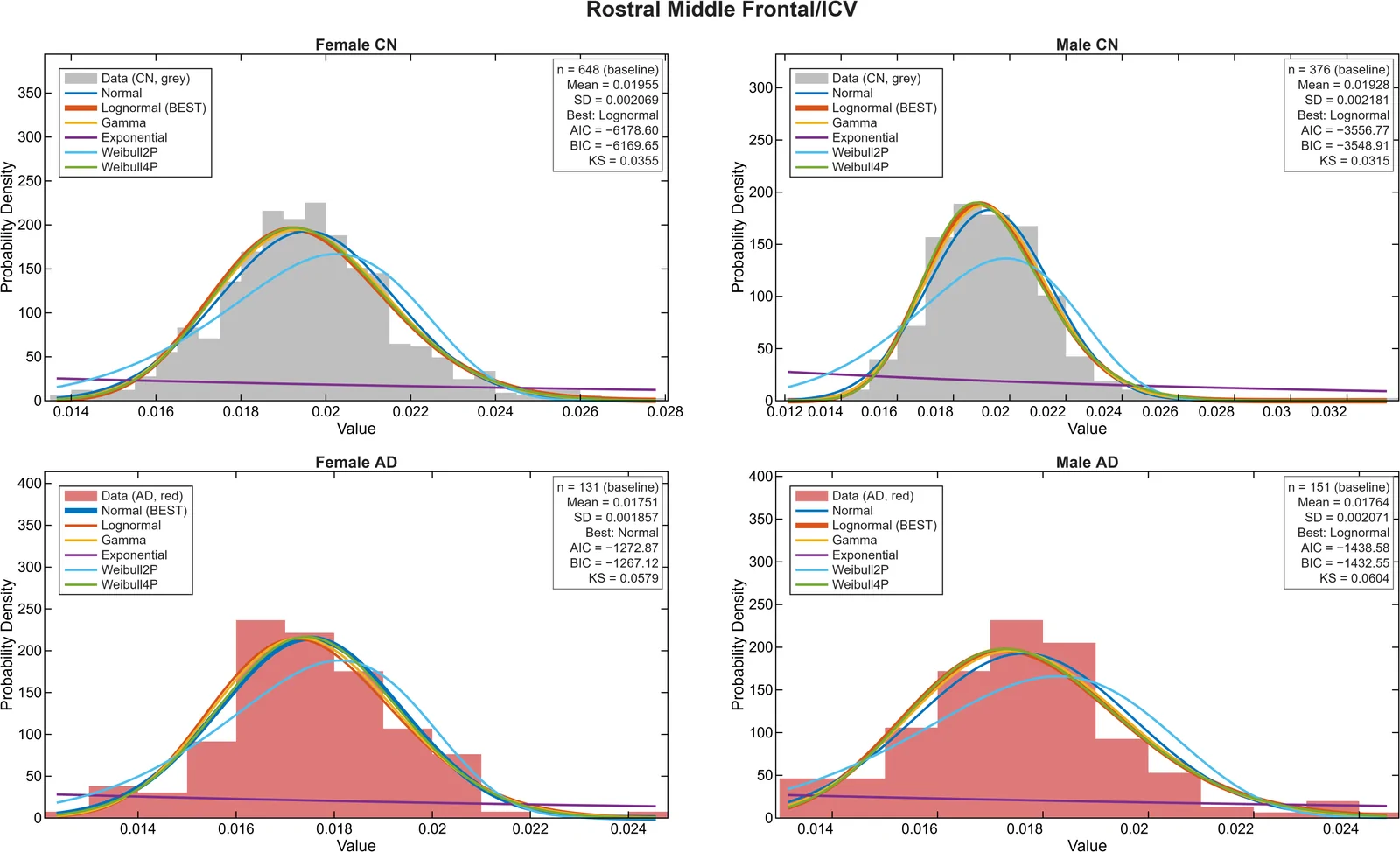
Rostral middle frontal gyrus volume/ICV—lowest AIC: Lognormal in Female-CN, Male-CN, and Male-AD; Normal in Female-AD. Weibull4P was adequate in all four groups (largest ΔAIC, 4.9).

**Figure A14:**
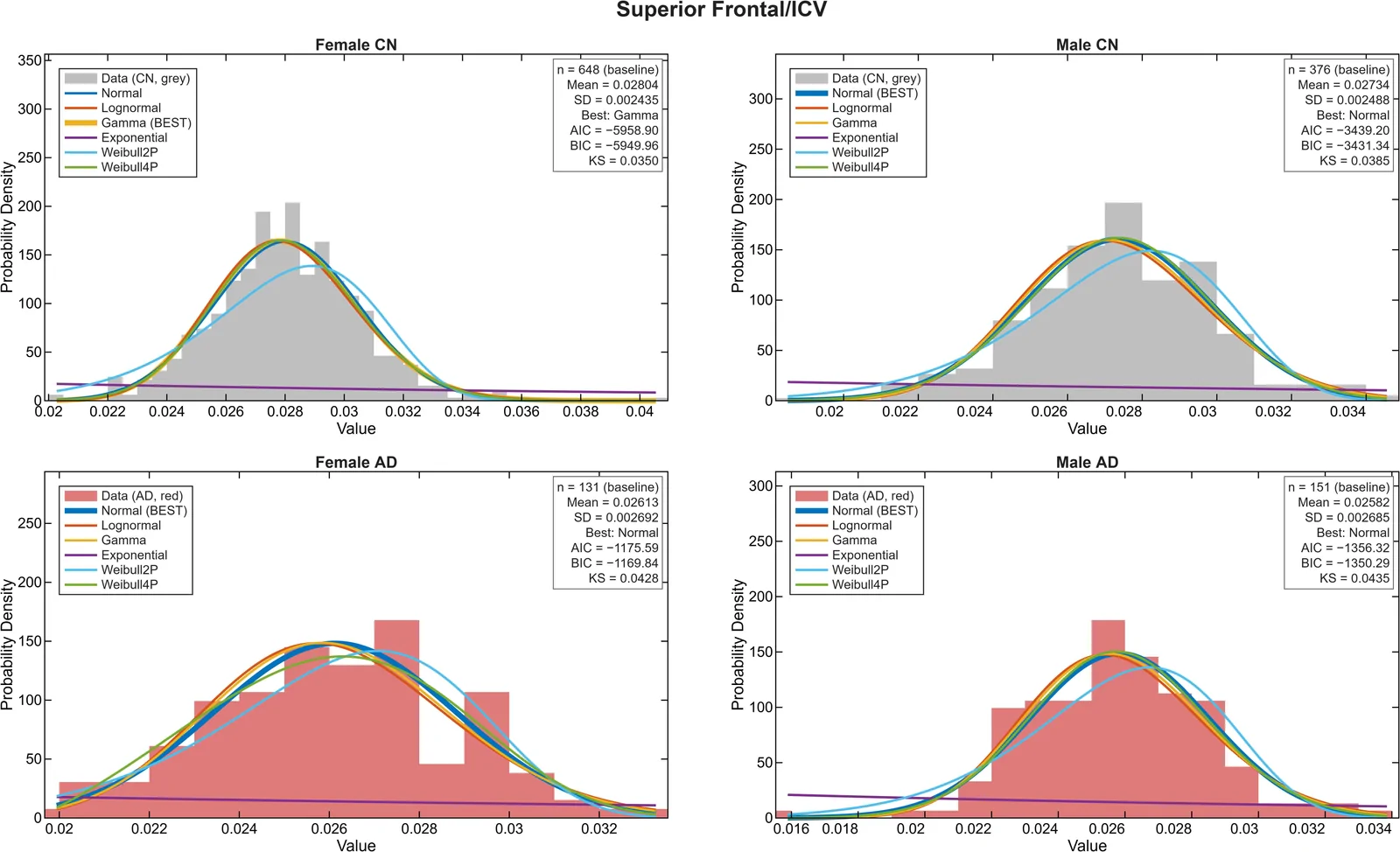
Superior frontal gyrus volume/ICV—lowest AIC: Gamma in Female-CN; Normal in Male-CN, Female-AD, and Male-AD. Weibull4P was adequate in all four groups (largest ΔAIC, 3.9).

**Figure A15:**
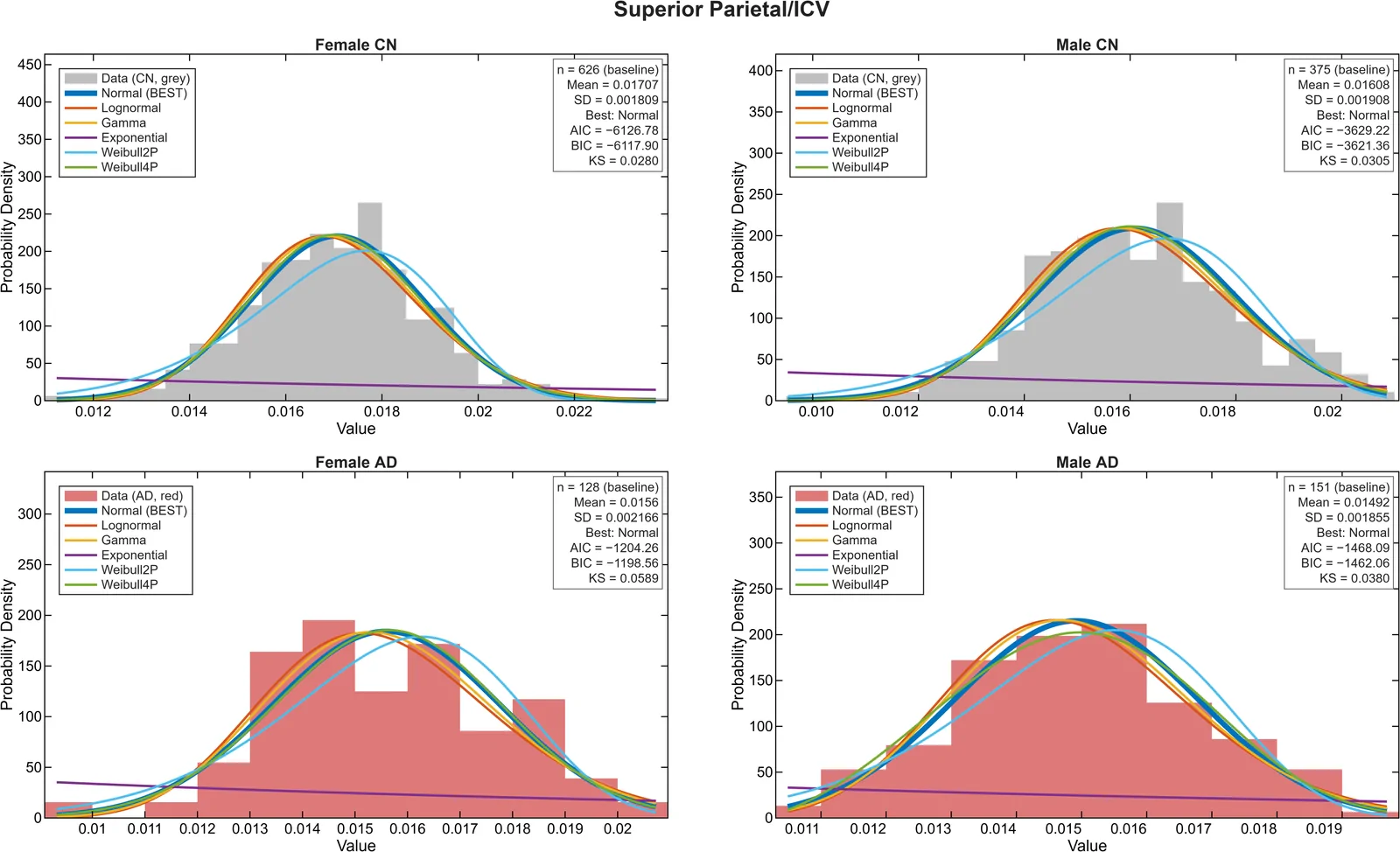
Superior parietal lobule volume/ICV—Normal had the lowest AIC in all four groups. Weibull4P was adequate in all four groups (largest ΔAIC, 3.9).

**Figure A16:**
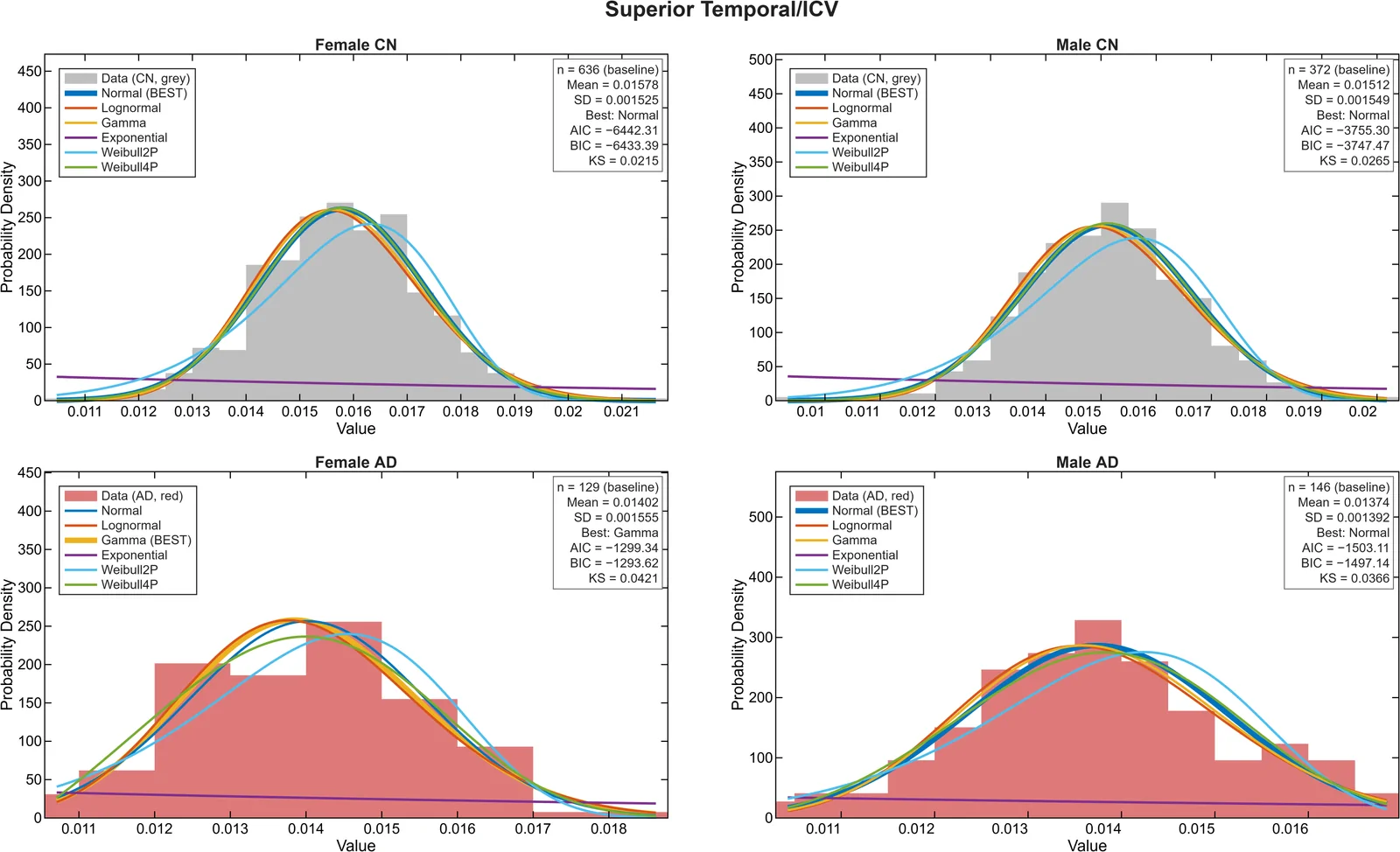
Superior temporal gyrus volume/ICV—lowest AIC: Normal in Female-CN, Male-CN, and Male-AD; Gamma in Female-AD. Weibull4P was adequate in all four groups (largest ΔAIC, 3.9).

**Figure A17:**
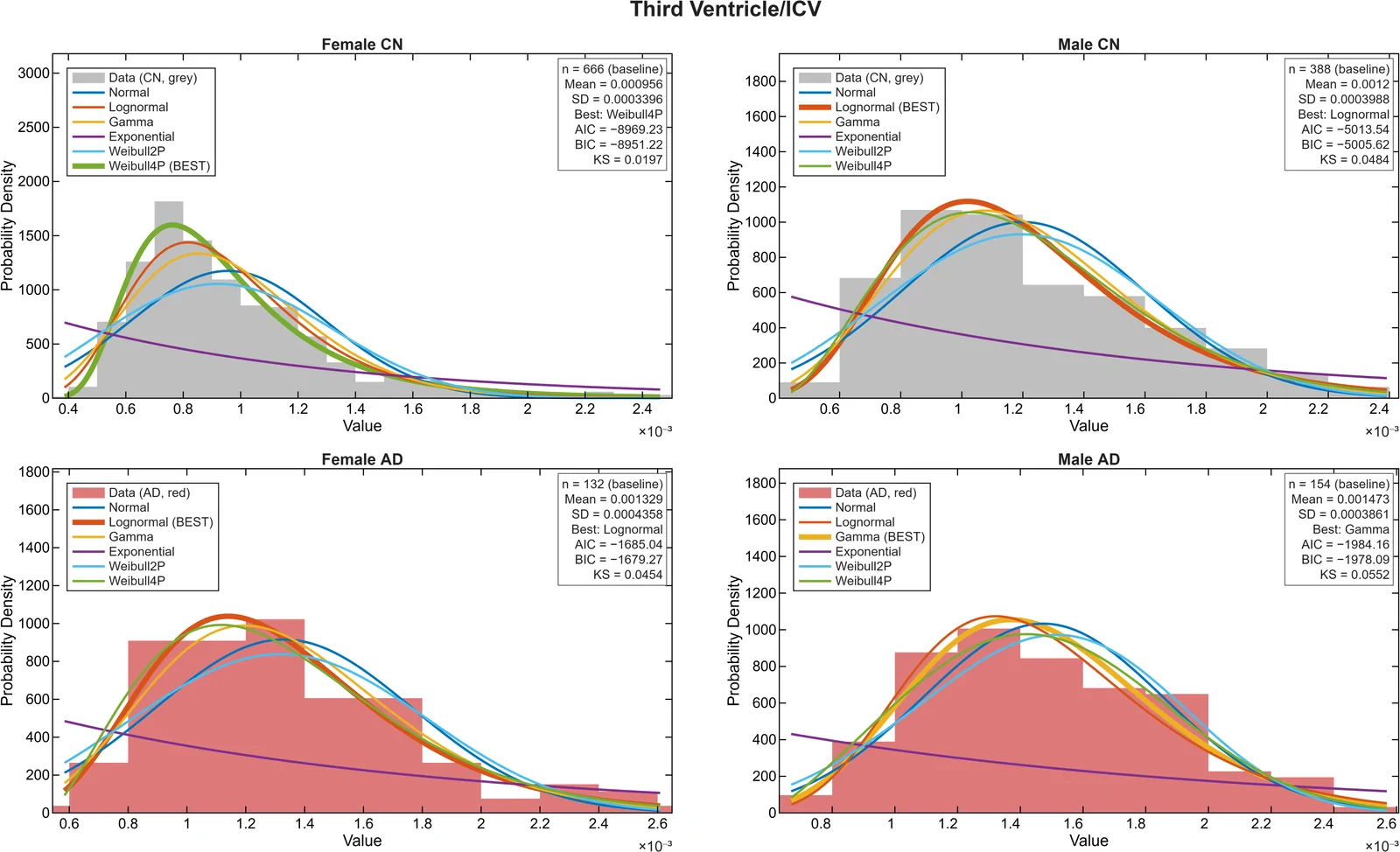
Third ventricle volume/ICV—lowest AIC: Weibull4P in Female-CN; Lognormal in Male-CN and Female-AD; Gamma in Male-AD. Weibull4P was adequate in all four groups (largest ΔAIC, 2.5).

**Figure A18:**
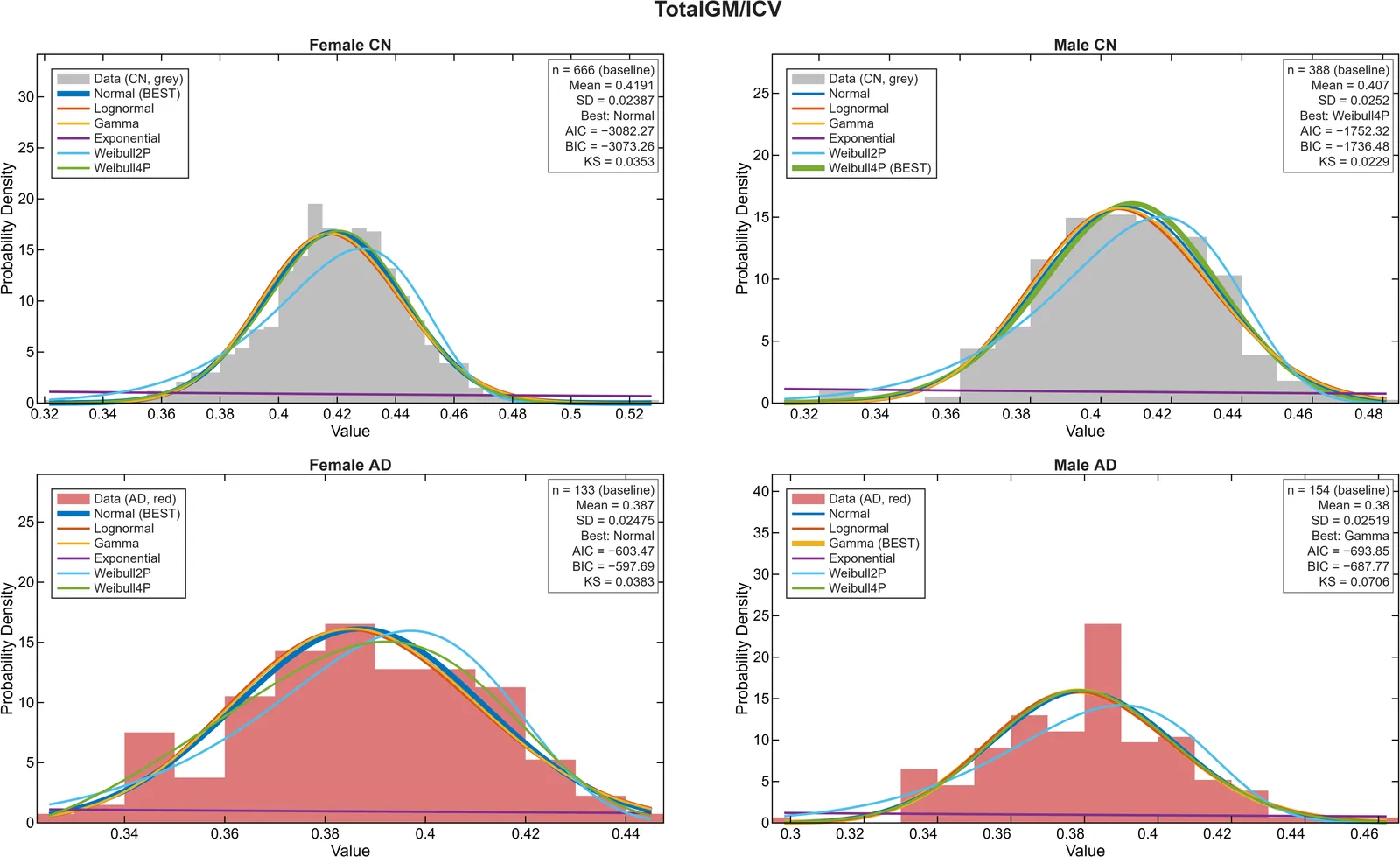
Total gray matter volume/ICV—lowest AIC: Normal in Female-CN and Female-AD; Weibull4P in Male-CN; Gamma in Male-AD. Weibull4P was adequate in all four groups (largest ΔAIC, 3.7).

**Figure A19:**
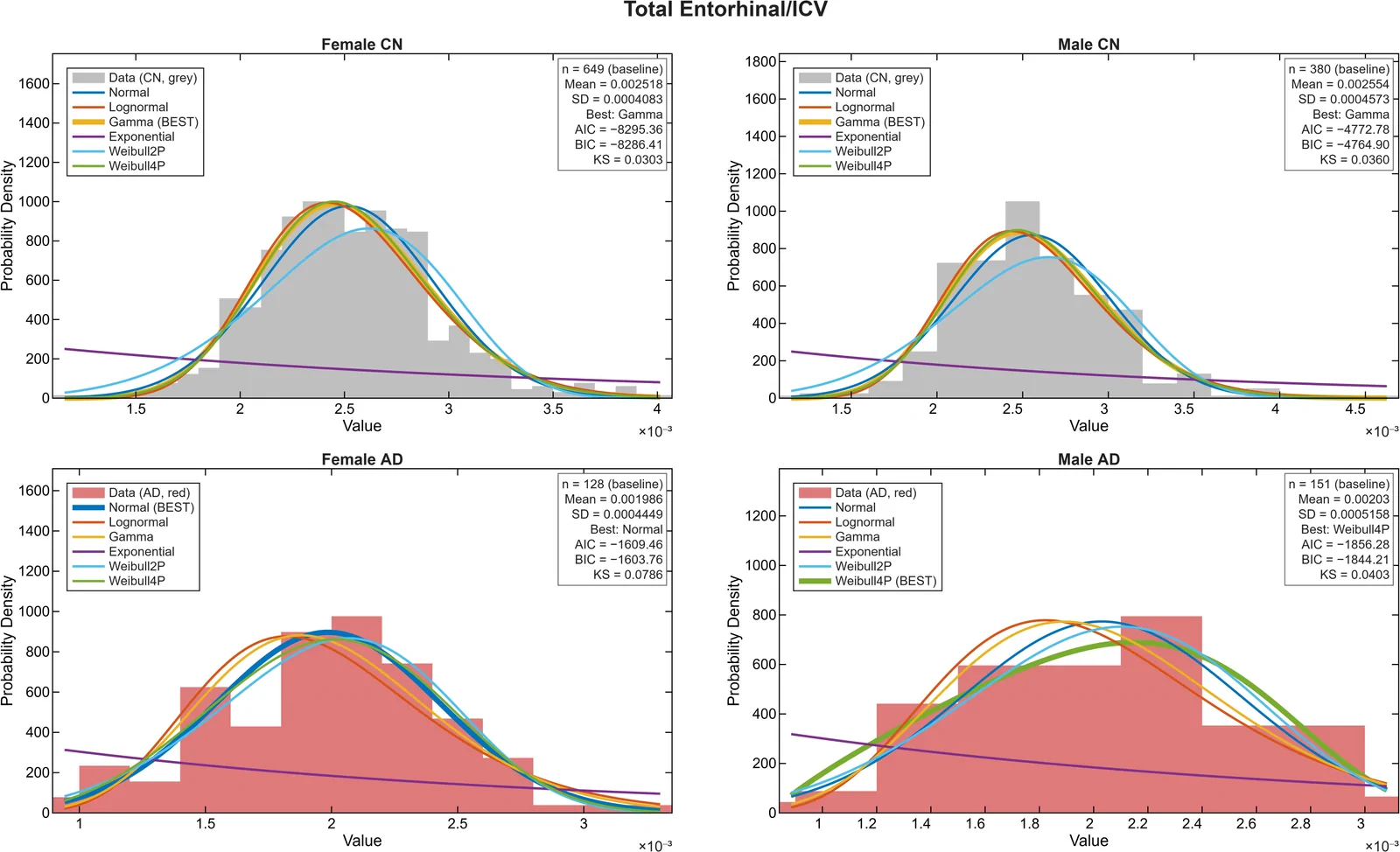
Total entorhinal cortex volume/ICV—lowest AIC: Gamma in Female-CN and Male-CN; Normal in Female-AD; Weibull4P in Male-AD. Weibull4P was adequate in all four groups (largest ΔAIC, 3.3).

**Figure A20:**
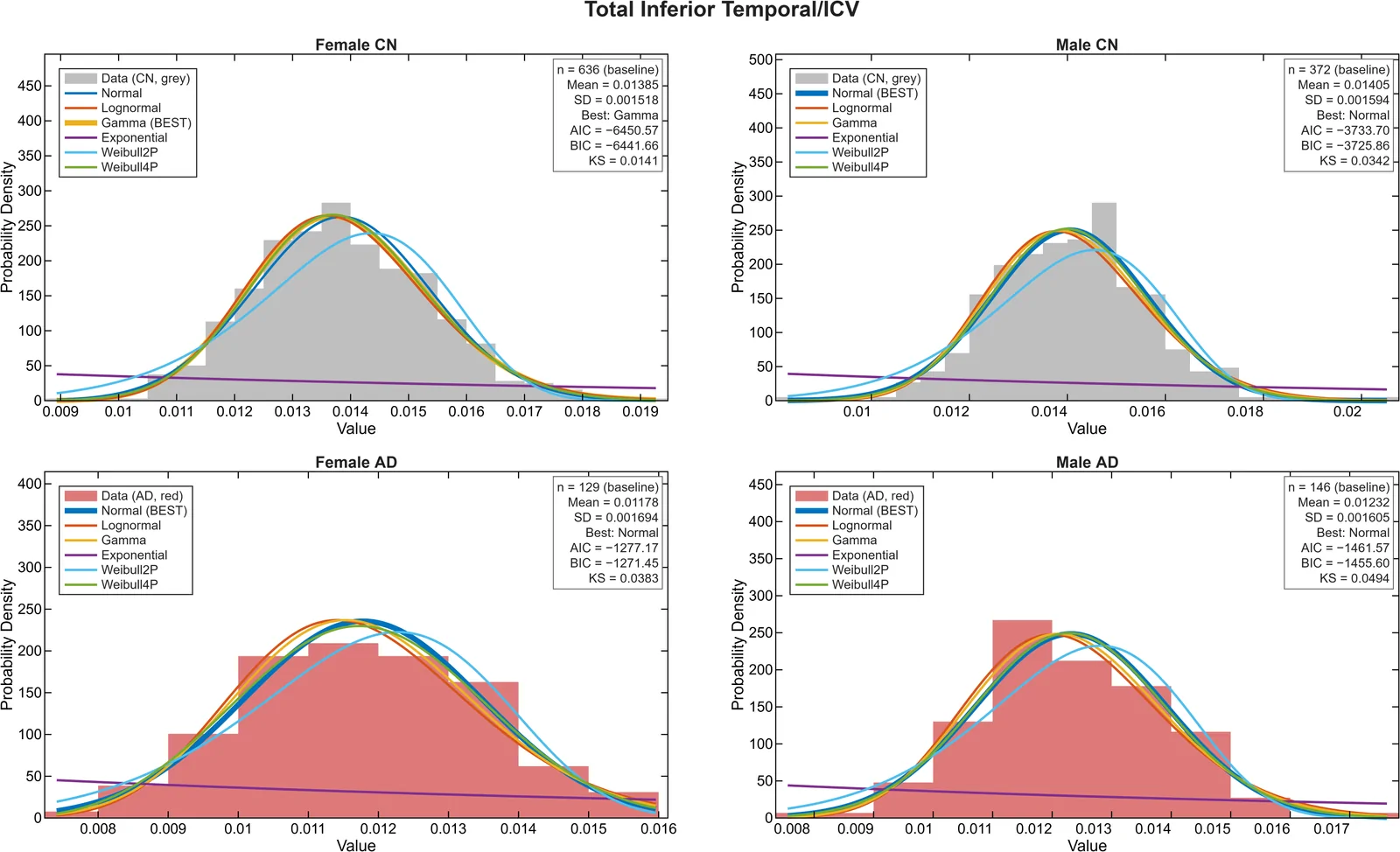
Total inferior temporal gyrus volume/ICV—lowest AIC: Gamma in Female-CN; Normal in Male-CN, Female-AD, and Male-AD. Weibull4P was adequate in all four groups (largest ΔAIC, 4.1).

## Data Availability

Individual participant-level data cannot be redistributed by the authors under the ADNI data use agreement. The region-by-group results supporting the findings of this study are provided in Supplementary Table S1.

## Supplementary Materials

The following supporting information can be downloaded at: https://www.mdpi.com/article/10.3390/life16101741/s1, Table S1: Region-by-group results: ICV quality control and sample, distribution fitting and model selection (AIC, BIC, Vuong tests), Weibull4P estimation and identifiability, normality tests, parametric-bootstrap goodness of fit, participant-bootstrap stability, cross-validation, age-stratified refits and AD–CN comparisons (Excel workbook).

## Abbreviations

AD	Alzheimer’s disease
ADNI	Alzheimer’s Disease Neuroimaging Initiative
AIC	Akaike Information Criterion
ANCOVA	Analysis of Covariance
BIC	Bayesian Information Criterion
CN	Cognitively Normal
CV	Cross-Validation
FDR	False Discovery Rate
GM	Gray Matter
ICV	Intracranial Volume
KS	Kolmogorov–Smirnov
MCI	Mild Cognitive Impairment
MRI	Magnetic Resonance Imaging
PET	Positron Emission Tomography
RMSE	Root Mean Square Error
W2P	Two-parameter Weibull distribution
W4P	Four-parameter Exponentiated Weibull distribution (Weibull4P)
WM	White Matter

## Appendix B. Goodness-of-Fit and Stability Diagnostics for All 84 Region × Group Combinations

Table A1 reports, for every one of the 84 region-by-group combinations, the distribution with the lowest stability-qualified AIC in the baseline data (one scan per participant) and its diagnostics: baseline sample size (N), the lowest-AIC distribution, AIC, BIC, the observed Kolmogorov–Smirnov statistic, the parametric-bootstrap Anderson–Darling *p*-value of the lowest-AIC distribution (Section 2.6), and the baseline participant-bootstrap selection frequency, that is, the percentage of the 500 replicates in which the same distribution had the lowest AIC, together with the distribution that most frequently had the lowest AIC (Section 2.6). The goodness-of-fit results for all six distributions are given in Supplementary Table S1. Regions are ordered alphabetically as in Table 4; within each region, groups are ordered Female-CN, Male-CN, Female-AD, Male-AD.

**Table A1:** Goodness-of-fit and participant-bootstrap diagnostics for the distribution with the lowest stability-qualified AIC in each of the 84 region-by-group combinations (baseline).

Region	Group	Lowest AIC	N	AIC	BIC	KS stat.	Bootstrap AD *p*	Reproduced (%)	Most Frequent Participant Bootstrap
Amygdala	Female-CN	Normal	649	−8734.9	−8726.0	0.0301	0.128	85.0	Same
Amygdala	Male-CN	Normal	380	−5077.8	−5069.9	0.0355	0.271	71.6	Same
Amygdala	Female-AD	Gamma	128	−1681.3	−1675.6	0.0507	0.653	38.6	Lognormal
Amygdala	Male-AD	Lognormal	151	−1985.2	−1979.2	0.0459	0.790	61.4	Same
Anterior Cingulate	Female-CN	Weibull4P	666	−7716.9	−7698.9	0.0314	0.020	32.4	Normal
Anterior Cingulate	Male-CN	Gamma	388	−4510.5	−4502.6	0.0385	0.058	51.0	Same
Anterior Cingulate	Female-AD	Normal	133	−1554.8	−1549.0	0.0557	0.719	42.8	Weibull2P
Anterior Cingulate	Male-AD	Gamma	154	−1776.5	−1770.5	0.0523	0.188	33.2	Lognormal
Cortical GM	Female-CN	Normal	666	−3362.8	−3353.8	0.0397	0.004	71.2	Same
Cortical GM	Male-CN	Weibull4P	388	−1914.8	−1899.0	0.0394	0.056	54.6	Same
Cortical GM	Female-AD	Normal	133	−653.0	−647.3	0.0623	0.234	41.6	Weibull4P
Cortical GM	Male-AD	Normal	154	−756.4	−750.4	0.0641	0.353	47.6	Same
Cortical WM	Female-CN	Lognormal	662	−3148.1	−3139.1	0.0303	0.086	55.2	Same
Cortical WM	Male-CN	Normal	387	−1821.5	−1813.6	0.0251	0.705	76.8	Same
Cortical WM	Female-AD	Weibull2P	133	−576.8	−571.1	0.0557	0.126	82.0	Same
Cortical WM	Male-AD	Normal	154	−667.1	−661.0	0.0361	0.974	63.0	Same
Fusiform	Female-CN	Normal	621	−6591.3	−6582.5	0.0207	0.529	60.2	Same
Fusiform	Male-CN	Normal	366	−3824.8	−3817.0	0.0357	0.303	65.0	Same
Fusiform	Female-AD	Normal	127	−1308.3	−1302.6	0.0637	0.116	53.6	Same
Fusiform	Male-AD	Normal	144	−1480.6	−1474.6	0.0430	0.792	55.6	Same
Hippocampus	Female-CN	Weibull4P	649	−7932.8	−7914.9	0.0207	0.369	42.6	Same
Hippocampus	Male-CN	Normal	381	−4602.2	−4594.4	0.0260	0.737	85.2	Same
Hippocampus	Female-AD	Gamma	129	−1513.6	−1507.9	0.0410	0.872	31.6	Weibull4P
Hippocampus	Male-AD	Gamma	151	−1804.2	−1798.2	0.0468	0.259	37.4	Same
Inferior Lateral Ventricle	Female-CN	Weibull4P	666	−8952.1	−8934.1	0.0211	0.858	71.6	Same
Inferior Lateral Ventricle	Male-CN	Weibull4P	388	−4902.6	−4886.8	0.0270	0.481	64.2	Same
Inferior Lateral Ventricle	Female-AD	Lognormal	132	−1509.3	−1503.6	0.0480	0.313	66.8	Same
Inferior Lateral Ventricle	Male-AD	Gamma	154	−1721.4	−1715.4	0.0653	0.315	54.6	Same
Inferior Parietal	Female-CN	Gamma	626	−6100.7	−6091.9	0.0233	0.431	49.8	Same
Inferior Parietal	Male-CN	Gamma	375	−3634.3	−3626.4	0.0351	0.271	40.2	Same
Inferior Parietal	Female-AD	Normal	128	−1188.4	−1182.7	0.0453	0.924	48.0	Same
Inferior Parietal	Male-AD	Normal	151	−1472.0	−1466.0	0.0288	0.966	46.2	Same
Lateral Ventricle	Female-CN	Weibull4P	666	−4594.2	−4576.2	0.0292	0.261	78.8	Same
Lateral Ventricle	Male-CN	Lognormal	388	−2547.1	−2539.2	0.0365	0.407	53.2	Same
Lateral Ventricle	Female-AD	Gamma	132	−818.3	−812.5	0.0564	0.707	55.8	Same
Lateral Ventricle	Male-AD	Gamma	154	−950.8	−944.7	0.0470	0.593	61.4	Same
Middle Temporal	Female-CN	Normal	636	−6480.3	−6471.4	0.0231	0.337	45.0	Same
Middle Temporal	Male-CN	Lognormal	372	−3768.1	−3760.3	0.0296	0.541	49.2	Same
Middle Temporal	Female-AD	Normal	129	−1292.7	−1287.0	0.0566	0.852	45.6	Same
Middle Temporal	Male-AD	Lognormal	146	−1497.7	−1491.7	0.0419	0.655	56.0	Same
Parahippocampal	Female-CN	Gamma	649	−8526.8	−8517.9	0.0182	0.621	67.4	Same
Parahippocampal	Male-CN	Lognormal	380	−4872.5	−4864.6	0.0575	0.002	58.2	Same
Parahippocampal	Female-AD	Normal	128	−1647.7	−1642.0	0.0574	0.385	55.2	Same
Parahippocampal	Male-AD	Gamma	151	−1936.1	−1930.0	0.0359	0.950	38.8	Same
Posterior Cingulate	Female-CN	Normal	666	−8421.8	−8412.8	0.0286	0.307	78.2	Same
Posterior Cingulate	Male-CN	Normal	388	−4882.2	−4874.3	0.0324	0.359	81.4	Same
Posterior Cingulate	Female-AD	Weibull4P	133	−1650.9	−1639.3	0.0645	0.026	12.6	Weibull2P
Posterior Cingulate	Male-AD	Normal	154	−1903.9	−1897.8	0.0471	0.495	63.4	Same
Precuneus	Female-CN	Gamma	666	−7083.1	−7074.1	0.0214	0.671	60.2	Same
Precuneus	Male-CN	Normal	388	−4086.7	−4078.8	0.0246	0.960	69.4	Same
Precuneus	Female-AD	Gamma	133	−1359.3	−1353.5	0.0535	0.253	28.4	Lognormal
Precuneus	Male-AD	Normal	154	−1636.4	−1630.3	0.0598	0.373	59.2	Same
Rostral Middle Frontal	Female-CN	Lognormal	648	−6178.6	−6169.7	0.0355	0.010	56.8	Same
Rostral Middle Frontal	Male-CN	Lognormal	376	−3556.8	−3548.9	0.0315	0.242	51.6	Same
Rostral Middle Frontal	Female-AD	Normal	131	−1272.9	−1267.1	0.0579	0.230	52.0	Same
Rostral Middle Frontal	Male-AD	Lognormal	151	−1438.6	−1432.5	0.0604	0.128	60.6	Same
Superior Frontal	Female-CN	Gamma	648	−5958.9	−5950.0	0.0350	0.026	34.0	Normal
Superior Frontal	Male-CN	Normal	376	−3439.2	−3431.3	0.0385	0.126	85.2	Same
Superior Frontal	Female-AD	Normal	131	−1175.6	−1169.8	0.0428	0.886	36.4	Same
Superior Frontal	Male-AD	Normal	151	−1356.3	−1350.3	0.0435	0.517	53.0	Same
Superior Parietal	Female-CN	Normal	626	−6126.8	−6117.9	0.0280	0.397	68.8	Same
Superior Parietal	Male-CN	Normal	375	−3629.2	−3621.4	0.0305	0.128	64.4	Same
Superior Parietal	Female-AD	Normal	128	−1204.3	−1198.6	0.0589	0.214	58.8	Same
Superior Parietal	Male-AD	Normal	151	−1468.1	−1462.1	0.0380	0.968	40.0	Same
Superior Temporal	Female-CN	Normal	636	−6442.3	−6433.4	0.0215	0.673	79.6	Same
Superior Temporal	Male-CN	Normal	372	−3755.3	−3747.5	0.0265	0.545	80.2	Same
Superior Temporal	Female-AD	Gamma	129	−1299.3	−1293.6	0.0421	0.707	24.8	Normal
Superior Temporal	Male-AD	Normal	146	−1503.1	−1497.1	0.0366	0.727	68.2	Same
Third Ventricle	Female-CN	Weibull4P	666	−8969.2	−8951.2	0.0197	0.377	72.6	Same
Third Ventricle	Male-CN	Lognormal	388	−5013.5	−5005.6	0.0484	0.016	39.0	Weibull4P
Third Ventricle	Female-AD	Lognormal	132	−1685.0	−1679.3	0.0454	0.786	68.0	Same
Third Ventricle	Male-AD	Gamma	154	−1984.2	−1978.1	0.0552	0.589	54.8	Same
Total GM	Female-CN	Normal	666	−3082.3	−3073.3	0.0353	0.006	69.2	Same
Total GM	Male-CN	Weibull4P	388	−1752.3	−1736.5	0.0229	0.202	23.2	Normal
Total GM	Female-AD	Normal	133	−603.5	−597.7	0.0383	0.770	43.6	Same
Total GM	Male-AD	Gamma	154	−693.8	−687.8	0.0706	0.323	14.4	Normal
Total Entorhinal	Female-CN	Gamma	649	−8295.4	−8286.4	0.0303	0.096	49.4	Same
Total Entorhinal	Male-CN	Gamma	380	−4772.8	−4764.9	0.0360	0.114	48.8	Same
Total Entorhinal	Female-AD	Normal	128	−1609.5	−1603.8	0.0786	0.120	48.8	Same
Total Entorhinal	Male-AD	Weibull4P	151	−1856.3	−1844.2	0.0403	0.150	28.6	Weibull2P
Total Inferior Temporal	Female-CN	Gamma	636	−6450.6	−6441.7	0.0141	0.990	52.4	Same
Total Inferior Temporal	Male-CN	Normal	372	−3733.7	−3725.9	0.0342	0.242	70.6	Same
Total Inferior Temporal	Female-AD	Normal	129	−1277.2	−1271.4	0.0383	0.986	39.0	Same
Total Inferior Temporal	Male-AD	Normal	146	−1461.6	−1455.6	0.0494	0.735	51.0	Same
